# Thermal Stabilization of Dihydrofolate Reductase Using Monte Carlo Unfolding Simulations and Its Functional Consequences

**DOI:** 10.1371/journal.pcbi.1004207

**Published:** 2015-04-23

**Authors:** Jian Tian, Jaie C. Woodard, Andrew Whitney, Eugene I. Shakhnovich

**Affiliations:** 1 Department of Chemistry and Chemical Biology, Harvard University, Cambridge, Massachusetts, United States of America; 2 Biotechnology Research Institute, Chinese Academy of Agricultural Sciences, Beijing, China; 3 Graduate Program in Biophysics, Harvard University, Cambridge, Massachusetts, United States of America; University of Houston, United States of America

## Abstract

Design of proteins with desired thermal properties is important for scientific and biotechnological applications. Here we developed a theoretical approach to predict the effect of mutations on protein stability from non-equilibrium unfolding simulations. We establish a relative measure based on apparent simulated melting temperatures that is independent of simulation length and, under certain assumptions, proportional to equilibrium stability, and we justify this theoretical development with extensive simulations and experimental data. Using our new method based on all-atom Monte-Carlo unfolding simulations, we carried out a saturating mutagenesis of Dihydrofolate Reductase (DHFR), a key target of antibiotics and chemotherapeutic drugs. The method predicted more than 500 stabilizing mutations, several of which were selected for detailed computational and experimental analysis. We find a highly significant correlation of *r* = 0.65–0.68 between predicted and experimentally determined melting temperatures and unfolding denaturant concentrations for WT DHFR and 42 mutants. The correlation between energy of the native state and experimental denaturation temperature was much weaker, indicating the important role of entropy in protein stability. The most stabilizing point mutation was D27F, which is located in the active site of the protein, rendering it inactive. However for the rest of mutations outside of the active site we observed a weak yet statistically significant *positive* correlation between thermal stability and catalytic activity indicating the lack of a stability-activity tradeoff for DHFR. By combining stabilizing mutations predicted by our method, we created a highly stable catalytically active *E*. *coli* DHFR mutant with measured denaturation temperature 7.2°C higher than WT. Prediction results for DHFR and several other proteins indicate that computational approaches based on unfolding simulations are useful as a general technique to discover stabilizing mutations.

## Author Summary

All-atom molecular simulations have provided valuable insight into the workings of molecular machines and the folding and unfolding of proteins. However, commonly employed molecular dynamics simulations suffer from a limitation in accessible time scale, making it difficult to model large-scale unfolding events in a realistic amount of simulation time without employing unrealistically high temperatures. Here, we describe a rapid all-atom Monte Carlo simulation approach to simulate unfolding of the essential bacterial enzyme Dihydrofolate Reductase (DHFR) and all possible single point-mutants. We use these simulations to predict which mutants will be more thermodynamically stable (*i*.*e*., reside more often in the native folded state vs. the unfolded state) than the wild-type protein, and we confirm our predictions experimentally, creating several highly stable and catalytically active mutants. Thermally stable active engineered proteins can be used as a starting point in directed evolution experiments to evolve new functions on the background of this additional “reservoir of stability.” The stabilized enzyme may be able to accumulate a greater number of destabilizing yet functionally important mutations before unfolding, protease digestion, and aggregation abolish its activity.

## Introduction

Protein stability is an important determinant of organismal fitness and is central to the process of enzyme design for industrial applications [1–3]. Most proteins must be folded to carry out their functions *in vitro* or *in vivo*. In addition, non-functional aggregation of unfolded or partially-unfolded proteins can have a deleterious effect on the fitness of an organism and can lead to protein aggregation diseases, which include Alzheimer’s and Huntington’s, in humans [4–6]. Aggregation of poorly folded proteins can also hamper protein production for research and technological purposes [7].

While most mutations in a natural protein are destabilizing [8,9], biological proteins are not generally at their highest possible stability; some mutations will stabilize a protein, increasing the equilibrium population of the folded state [10–12]. This stabilization can be achieved by either slowing the rate of unfolding or speeding the rate of folding, depending on the role of the mutated residue in the folding nucleation process [13,14]. The unfolding temperature, *T*_m_, at which the free energy of the folded and unfolded states coincide (Δ*G* = 0) serves as a common measure of protein stability. *T*_m_ is obtainable by experiment and, in theory, from simulation, although current molecular dynamics simulations are limited in their ability to capture full folding or unfolding trajectories of most proteins (except very small fast folding domains [15]) in a tractable amount of simulation time [16].

Several computational methods to predict protein stability or changes in stability upon mutation have been developed and tested [17–19]. However, the performance of these popular methods is still relatively weak [20–22]. Other existing techniques to rationally design proteins with improved stability have involved optimization of charge-charge interactions [23], saturation mutagenesis of residues with high crystallographic B-factors [24], methods based on protein simulation and calculation of free energies [25–27] and comparison to homologous proteins including the ultra-stable proteins of thermophiles [28,29]. We reasoned that better predictions of mutant stability might be obtained by evaluating the unfolding temperature *T*_m_ in realistic yet computationally tractable simulations of protein unfolding.

Here, we use a Monte Carlo protein unfolding approach (MCPU) with an all-atom simulation method and knowledge-based potential developed earlier in our lab [16,30,31] to simulate unfolding and predict melting temperatures for all possible single point mutants of *E*. *coli* Dihydrofolate Reductase (DHFR). DHFR is an essential enzyme in bacteria and higher organisms, and it is an important target of antibiotics [32] and anti-cancer drugs [33,34]. Its moderate size (18 kDa) makes it amenable to both simulation and experiment. As described in the Materials and Methods section, the Monte Carlo move set consists of rotations about torsional angles. At high temperature, the higher entropy of unfolded states overcomes the increase in energy due to loss of favorable contacts and torsional preferences, leading to unfolding. We experimentally determine melting temperatures and catalytic activities for several predicted stabilizing mutants, and for mutants combining multiple stabilizing mutations. Our approach allows us to identify several stabilized mutants of DHFR, and our prediction method marks an improvement over existing stability predictors such as Eris [19], FoldX [17], and PopMusic [18]. Simulations of non-DHFR proteins likewise indicated that our method is useful as a general approach to simulate protein unfolding and select stabilizing mutations.

## Results

### Predicting the effects of mutations on protein stability from non-equilibrium unfolding simulations

Ideally, protein stability for any sequence should be predicted in all-atom equilibrium simulations that cover multiple folding-unfolding events to determine equilibrium populations of various states of the protein. However, despite recent progress in *ab initio* simulations of protein folding [15] this goal is not attainable for proteins of realistic size and biological relevance. Currently, non-equilibrium unfolding simulations are within reach for sufficiently large proteins and the question arises whether such simulations can be used to assess mutational effects on protein stability, which is an equilibrium property. The following analysis provides an affirmative answer to this question, under certain assumptions. Although the idea of obtaining equilibrium free energy differences from non-equilibrium measurements is not new [35], and protein stabilities have been calculated from molecular dynamics simulations using the Jarzynski equality, *e*.*g*., [36–38], such simulations require application of an external steering force; in the present paper we report the use of multi-temperature Monte-Carlo unfolding simulations in obtaining protein stabilities.

**Fig 1:**
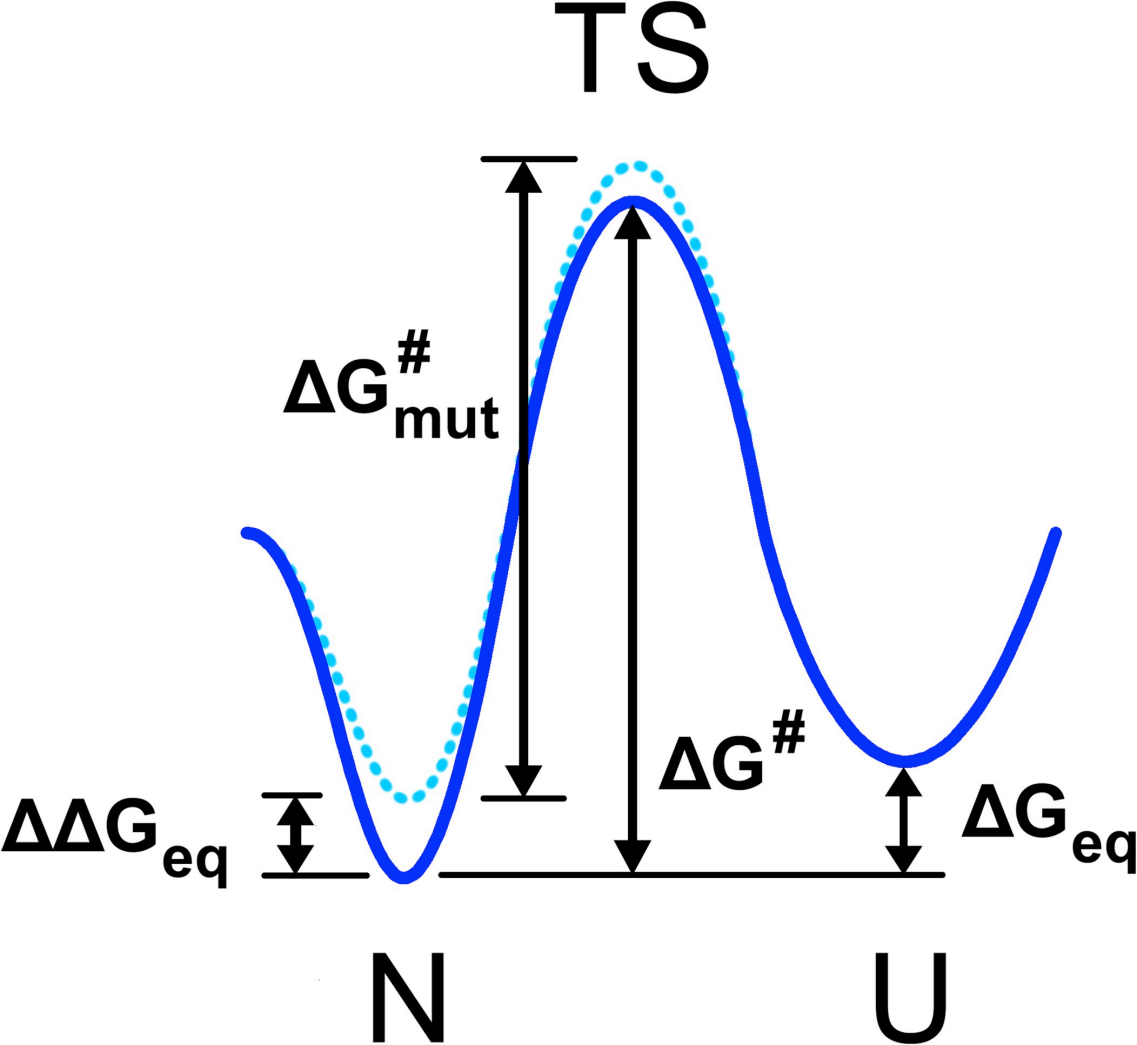
Two state diagram depicting the protein native state N, transition state TS, and unfolded state U. The WT energy landscape is shown in solid blue and the mutant energy landscape in dotted blue. The difference in free energy between WT folded and unfolded states, Δ*G*_*eq*_, the change in this quantity upon mutation, ΔΔ*G*_*eq*_, and the height of the transition state barrier relative to the native state for WT (Δ*G*^#^) and mutant ($\Delta G_{\text{mut}}^{\#}$) are labeled.

Assuming two-state unfolding kinetics [39–42] we can estimate the characteristic time required to cross the unfolding free energy barrier (in fact it is the time spent in the native state waiting for sufficient thermal fluctuation to cross the barrier) as:

$$\tau_{u}^{fp}=\tau_{0}e^{\frac{\Delta G^{\#}}{kT}}$$ (1)

where $\tau_{u}^{fp}$ is first-passage time from the folded to the unfolded state, Δ*G*^#^ is the free energy barrier between the folded state and the transition state for unfolding (see Fig. 1) and *τ*_0_ is the elementary time constant. When simulation time *τ*_*sim*_ approaches $\tau_{u}^{fp}$ unfolding events are observed in simulation. The apparent “melting temperature”, *i*.*e*., the temperature at which unfolding events occur in simulations, therefore depends on the simulation time *τ*_*sim*_ according to Eq. (1):

$$kT_{m}^{app}=\frac{\Delta G^{\#}}{\text{ln}\left(\frac{\tau_{sim}}{\tau_{0}}\right)}$$ (2)

This analysis suggests that non-equilibrium first passage unfolding simulations are not suitable to predict the temperature at which a protein would unfold at equilibrium. However the effect of mutations on stability can be predicted from unfolding simulations. In order to see this we note that the mutational effect on protein stability ΔΔ*G* is related to the change in the unfolding free energy barrier ΔΔ*G*^#^, the difference between the WT barrier height and the mutant barrier height, shown in Fig. 1.

$$\Delta \Delta G_{i}^{\#}=\left(1-\varphi_{i}\right)\Delta \Delta G_{i}^{eq}$$ (3)

where *i* denotes the mutated amino acid and *φ*_*i*_ is the *φ*-value for residue *i* which determines the fraction of interactions that this residue forms in the folding/unfolding transition state [40,43,44]. We therefore obtain

$$\;k\Delta T_{m}^{app}\left(i\right)=\frac{\left(1-\varphi_{i}\right)\Delta \Delta G_{i}^{eq}}{\text{ln}\left(\frac{\tau_{sim}}{\tau_{0}}\right)}$$ (4)

where $\Delta T_{m}^{app}\left(i\right)=T_{m}^{app}\left(i\right)-T_{m}^{app}\left(WT\right)$ is the shift in apparent unfolding temperature upon a specific mutation in the *i*-th residue. Introducing the relative (to WT) unfolding temperature $\Delta T_{m}^{rel}\left(i\right)=\Delta T_{m}^{app}\left(i\right)/T_{m}^{app}\left(WT\right)$ we get

$$\Delta T_{m}^{rel}\left(i\right)=\frac{\left(1-\varphi_{i}\right)\Delta \Delta G_{i}^{eq}}{\Delta G^{\#}}$$ (5)

*i*.*e*. the mutational shift in observed unfolding temperature, normalized to the observed unfolding temperature of the wild-type at the same simulation condition does not depend on the simulation length, provided that the simulation is sufficiently equilibrated *in the native basin* so that the rules of transition state theory apply. The analysis of extensive kinetic and equilibrium data for multiple proteins shows that for the majority of mutations (except for a small fraction of residues that participate in the folding nucleus) *φ*_*i*_ ≈ 0.24 with remarkable accuracy and consistency [45]. We get therefore

$$\Delta T_{m}^{rel}\left(i\right)=0.76\;\frac{\Delta \Delta G_{i}^{eq}}{\Delta G^{\#}}$$ (6)

*i*.*e*. $\Delta T_{m}^{rel}\left(i\right)$ is independent of simulation time and proportional to the equilibrium free energy effect of mutations, provided that simulations have equilibrated in the native basin of attraction.

### Monte Carlo protein unfolding simulation

**Fig 2:**
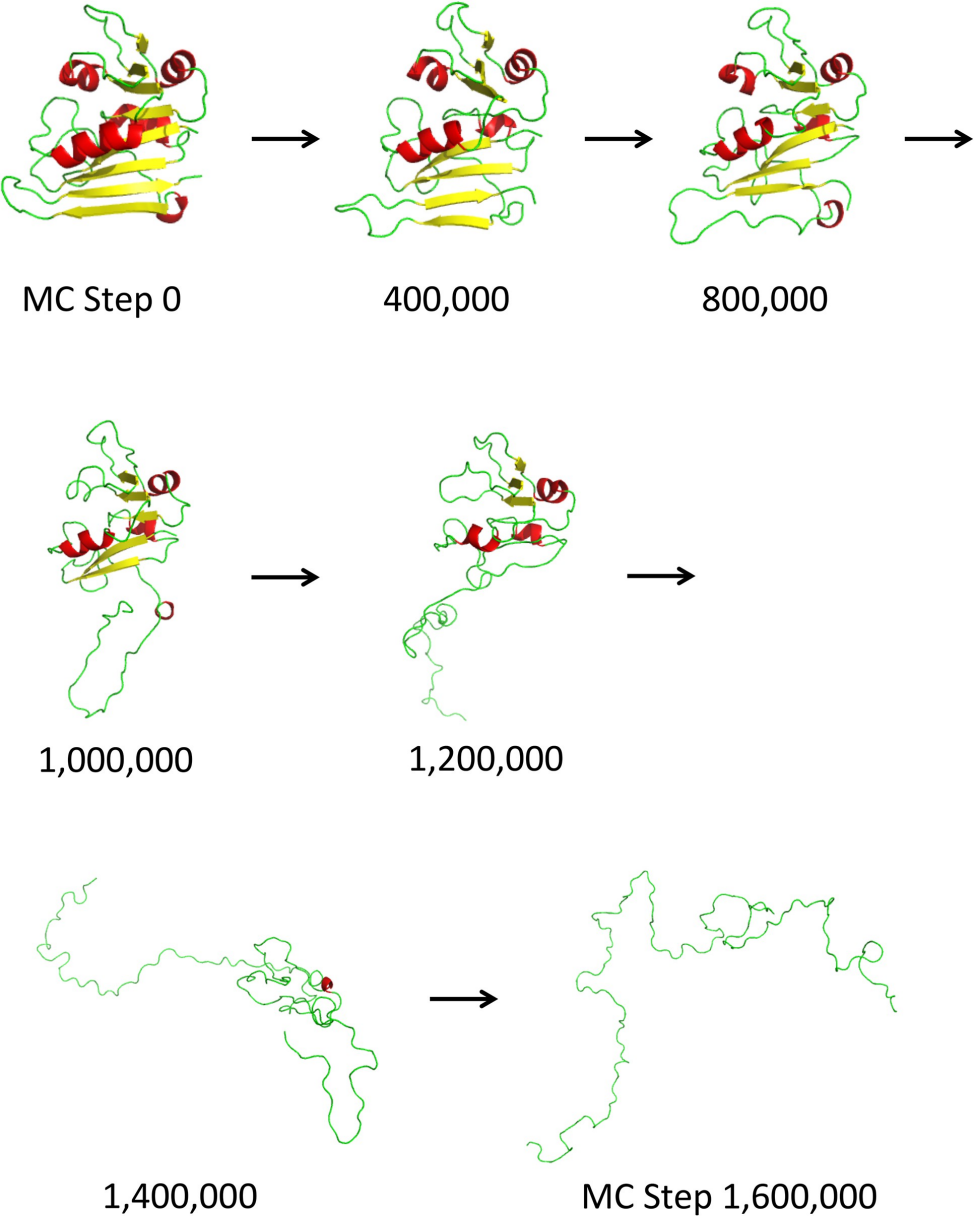
A sample WT DHFR unfolding trajectory at simulation temperature 1.5 (arbitrary simulation units). In MC simulations, separation of the C-terminal beta hairpin from the rest of the protein (steps 1,000,000 through 1,200,000) is an early event in the unfolding process.

**Fig 3:**
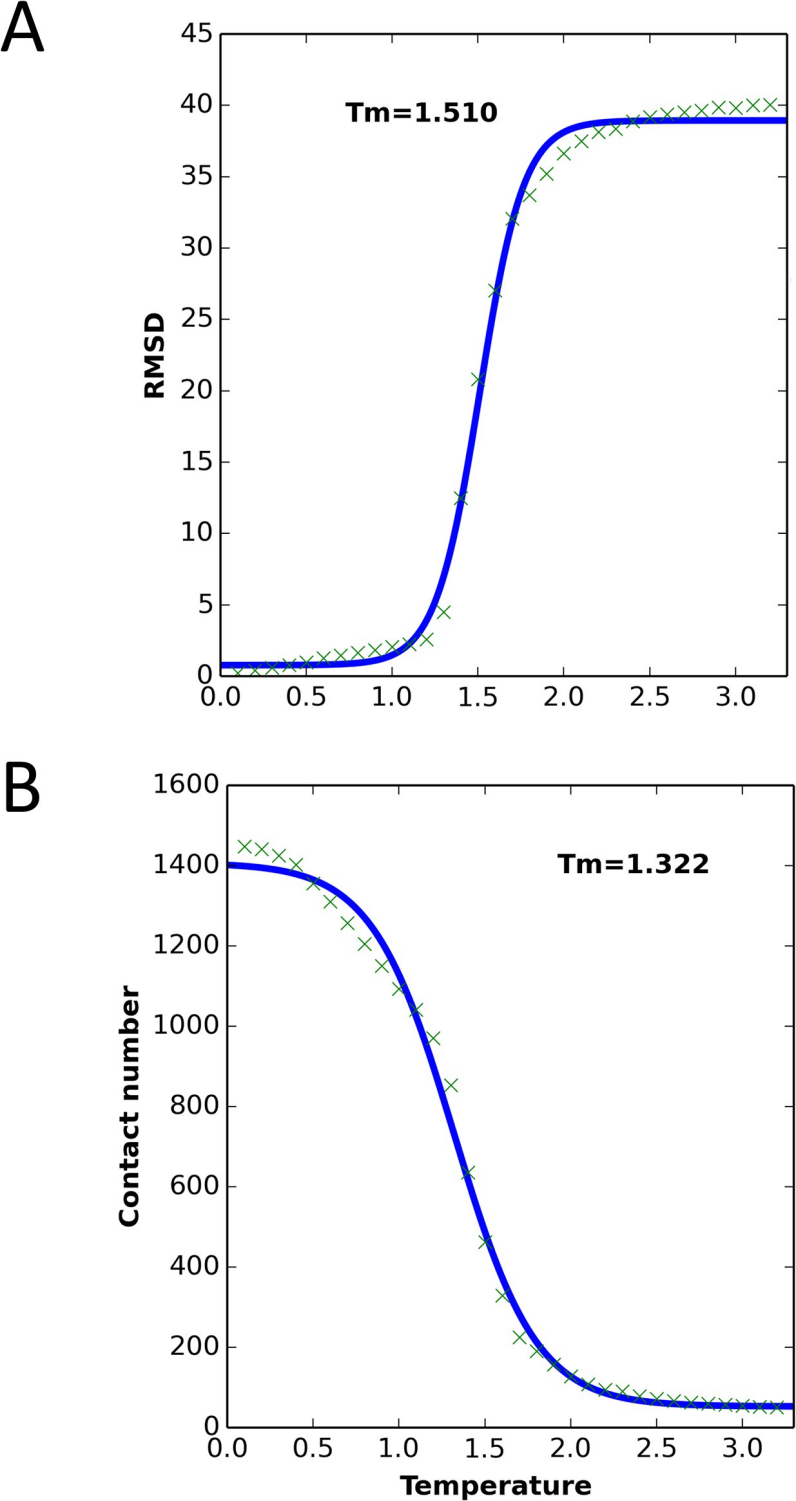
WT DHFR unfolding curves from MC simulations, averaged over 2,000,000 simulation steps, with 50 replications. The *T*_m_ value was calculated based on the sigmoidal fit (solid blue line). (A) RMSD vs. simulation temperature. (B) Number of contacts vs. simulation temperature.

We ran MCPU on DHFR (PDB ID: 4DFR) at a range of temperatures, to generate simulated unfolding curves. Unfolding steps of a sample trajectory are shown in Fig. 2, and a flowchart of the simulation method is shown in S1 Fig. The protein was subject to a brief MD energy minimization, beginning from the WT crystallographic native state, followed by unfolding simulations at each of 32 different temperatures using all-atom Monte-Carlo (see Materials and Methods section). As shown in figures S2 Fig—;S4 Fig, the RMSD and total energy increased and the number of contacts decreased as each simulation proceeded, and with increasing temperature. (Here, temperature is given in arbitrary simulation units.) Plots of RMSD and contact number vs. temperature showed sigmoidal behavior, with a clearly identifiable transition temperature, and the melting temperature (*T*_m_) could be determined by fitting to a sigmoidal function (Fig. 3). Plots of energy vs. temperature (S5 Fig) were sigmoid-like, but with an additional rise in energy at low to intermediate temperatures, perhaps indicating pre-melting to a dry-molten globule state with loosened side chains but native-like topology [46,47]. This deviation from sigmoidal behavior becomes clearer as the simulation length is increased (S6 Fig).

### Computational identification of stabilizing single point mutations

**Fig 4:**
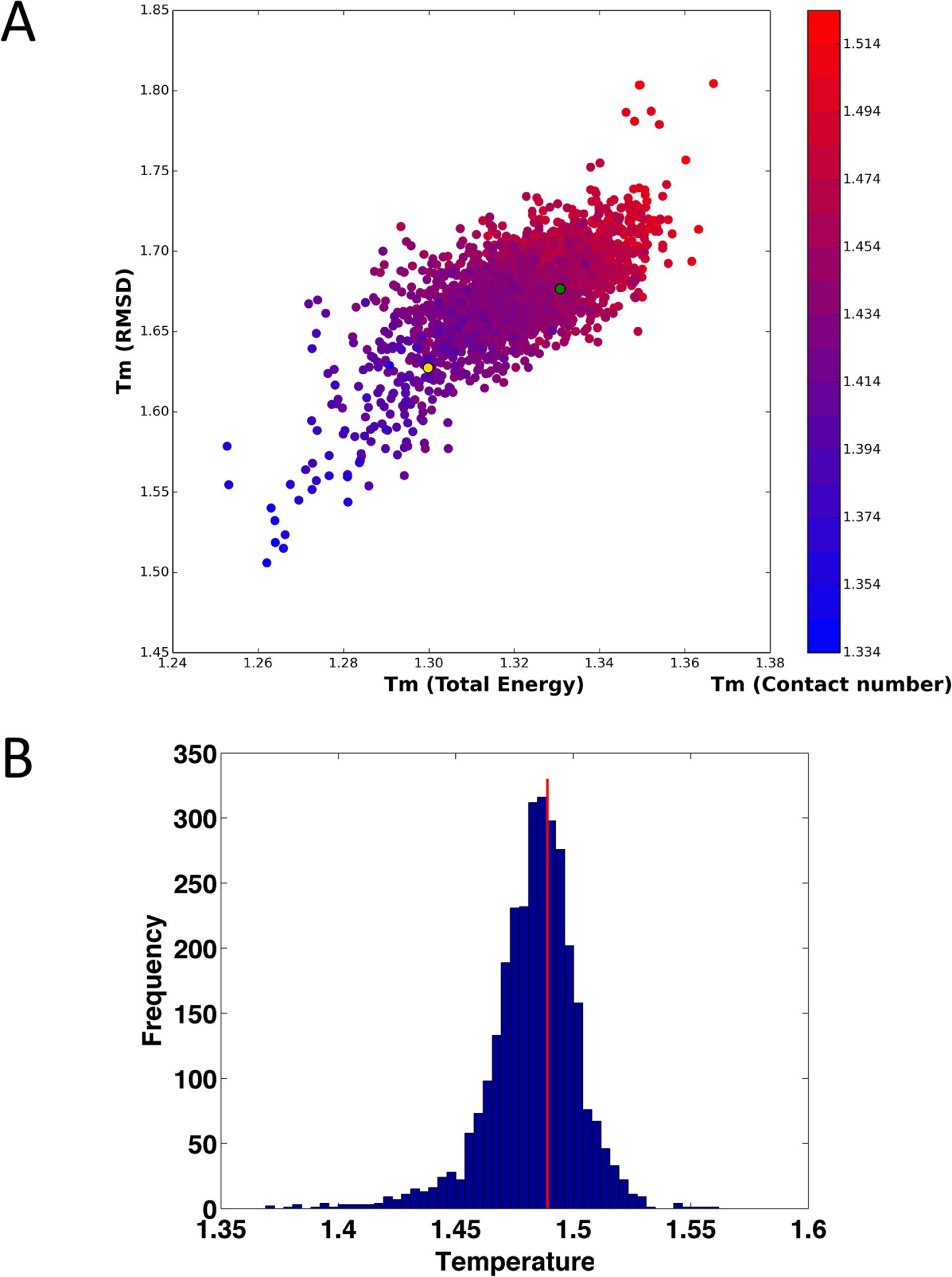
Simulated *T*_m_ values, based on RMSD, Total Energy and Contact number. (A) Scatter plot of *T*_m_ (RMSD) vs. *T*_m_ (Total energy), with *T*_m_ (contact number) represented by color (see color bar to right of plot). The green ball denotes WT and the gold ball denotes the destabilized mutant I155A. The correlation coefficients of simulated *T*_m_ between RMSD and total energy, RMSD and Contact number, and Contact number and total energy were 0.68, 0.79 and 0.84, respectively. (B) Histogram of *T*_m_ values, determined by averaging the values obtained from RMSD, energy, and contact number. The vertical red line denotes WT *T*_m._

**Fig 5:**
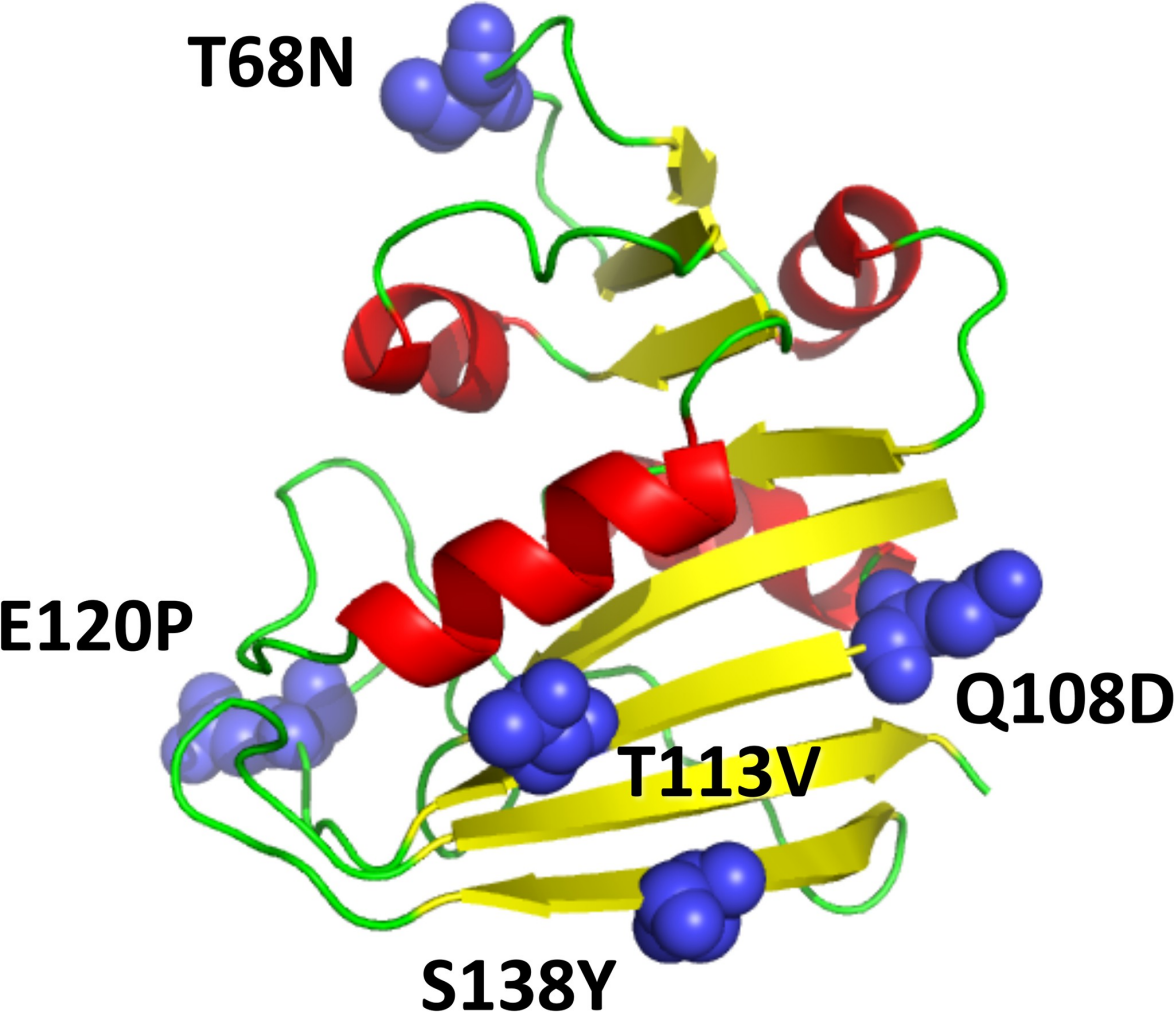
The 3D structure of DHFR (PDB ID 4DFR), with residues that were altered in the stabilized quintuple mutant shown in blue.

All possible single point mutations of DHFR (159 * 19 = 3,021) were simulated with the Monte Carlo protein unfolding simulation protocol. The simulated *T*_m_ values were calculated as described above. Of the 3,021 mutations, 523 mutations (17.3%) were predicted to have a stabilizing effect according to all three metrics (energy, contacts, and RMSD), while 42.1% of mutations had a destabilizing effect according to all three metrics. These predictions are in good agreement with statistical analysis of published experimental data and FoldX predictions [8,12]. The simulated *T*_m_ values evaluated using RMSD, total energy, and number of contacts are strongly correlated, as shown in Fig. 4A. The distribution of predicted melting temperatures (averaged over the 3 metrics) for all 3021 point mutants is shown in Fig. 4B. Next, we selected a subset of predicted stabilizing mutations for subsequent in depth computational and experimental analysis. To that end we selected the loci where multiple mutations were consistently predicted as stabilizing. Out of this set we selected one mutation at each loci which were predicted as most stabilizing. As a result we arrived at 23 single predicted stabilizing point mutants shown in S1 Table, which we deemed most promising for subsequent in depth computational and experimental analysis. Furthermore, five stabilizing mutations at different sites within DHFR, shown in Fig. 5, were combined to form the multiple mutants listed in Table 1, with the rationale that the combination of individual stabilizing mutants often yields more stable proteins, and these mutants were likewise subjected to computational and experimental analysis.

**Table 1:** The simulated and experimental results of the selected single point mutants and WT.

Mutation(s)	***T***_m_ (DSC)	***C***_m_ (CD)	***k***_cat_	***k***_cat_/***K***_m_	Simulated ***T***_m_
WT	54.1	3.09	24.60	14.07	1.358 ± 0.004
T113V	58.0	3.28	13.67	10.86	1.389 ± 0.004
Q108D	55.7	3.18	24.60	10.35	1.361 ± 0.004
S138Y	55.6	3.33	24.51	9.33	1.366 ± 0.004
D116F	55.5	3.43	24.80	9.53	1.369 ± 0.004
T68N	55.5	3.26	29.36	13.32	1.367 ± 0.003
E120P	55.3	3.25	30.02	13.91	1.371 ± 0.004
T68N,Q108D,T113V,E120P,S138Y	61.3	3.52	32.63	12.20	1.400 ± 0.004
T113V,E120P,S138Y	58.5	3.49	31.13	13.10	1.384 ± 0.004
T68N,Q108D,E120P,S138Y	56.4	3.47	22.80	10.94	1.377 ± 0.003
T68N,Q108D	55.8	3.14	17.99	15.24	1.366 ± 0.003
E120P,S138Y	55.6	3.29	16.01	10.81	1.371 ± 0.004

Note: The data were averaged over 50 replications. 2,000,000 MC steps were simulated in total, and the last 1,000,000 steps were used to calculate *T*_m_.

The units: *T*_m_: °C, *C*_m_: M, *k*_cat_: s^−1^, *k*_cat_∕*K*_M_: s^−1^ μM^−1^

### Computational test of the theoretical analysis

First we test two predictions that emerge from the theoretical analysis of unfolding simulations. The first prediction is that the apparent unfolding temperature decreases as the length of the unfolding simulation increases (Equation 4). Secondly and most importantly the mutational change in relative (normalized to WT) apparent unfolding temperature is a) robust with respect to simulation time provided that simulations have equilibrated in the native basin and b) directly proportional to the effect of mutations on equilibrium protein stability (Equation 6). We test these predictions using MCPU simulations and experiment.

**Fig 6:**
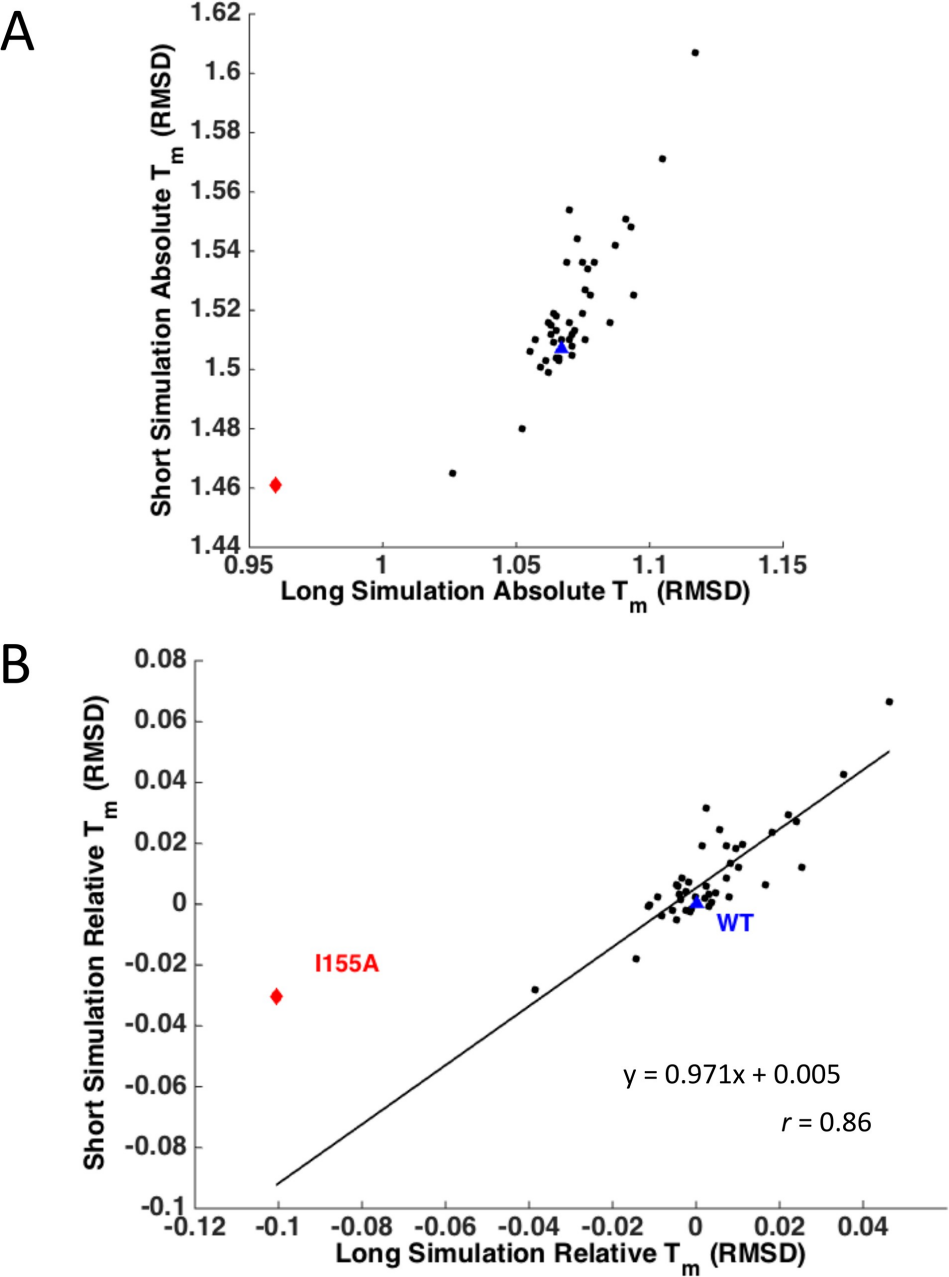
Correlation between *T*_m_ values for simulations of different lengths. WT is shown as a blue triangle and mutant I155A as a red diamond. (A) *T*_m_ calculated from simulation RMSD, for short (2,000,000-step) and long (20,000,000-step) simulations. Simulation *T*_m_ is clearly smaller for long simulations, in which the protein has more time to unfold. (B) Relative *T*_m_ normalized to WT, for short and long simulations. Remarkably, the points fall nearly on the line y = x, with a correlation of 0.86, with one distinct outlier I155A.

We carried out two sets of MCPU simulations of different lengths: 2,000,000 and 20,000,000 steps for the 23 predicted stabilizing mutants, 15 mutants studied previously by experiment [48] (the complete set of single mutants is listed in S1 Table), and the 5 stabilizing multiple mutants combining individual mutations listed in Table 1, and compared their predicted absolute and relative simulated unfolding temperatures (Fig. 6). Indeed both predictions of our theoretical analysis are confirmed, *i*.*e*., the apparent unfolding temperature decreases with simulation time (Fig. 6A) while the relative unfolding temperature $\Delta T_{m}^{rel}$ is remarkably independent of simulation time (Fig. 6B). We note that due to the nature of the energy function used in our simulations, there is no obvious mapping of simulation temperature to real absolute temperature (*i*.*e*., in Celsius or Kelvin). Conversion of simulation temperature to physical temperature would require use of experimental data (*e*.*g*., WT unfolding temperature and deviation of temperatures over all mutants) and therefore would not provide a completely simulation- or theory-based prediction. Furthermore, as noted above, the apparent absolute value of the transition temperature in the Monte-Carlo unfolding approach depends on simulation time. Therefore, we used relative melting temperature, $\Delta T_{m}^{rel}\left(i\right)=\Delta T_{m}^{app}\left(i\right)/T_{m}^{app}\left(WT\right)$, when comparing simulation results with experimental results.

As mentioned, the simulated *T*_m_ values evaluated using RMSD, total energy, and number of contacts are strongly correlated in our simulations as shown in Fig. 4A and S1 Table. In what follows we define the computational unfolding temperature *T*_m_ as averaged over *T*_m_ values determined using these three criteria.

### Experimental characterization of predicted mutants

We cloned, expressed, and purified the 23 single point mutants of DHFR listed in S1 Table, as well as the multiple mutants listed in Table 1 (see Materials and Methods). The biophysical properties of the mutants were measured and compared with WT DHFR, as shown in S2 Table. As many studies have shown that oligomerization can alter protein stability [23,48,49], we first tested whether mutations induce oligomerization and/or aggregation using the gel filtration method [48,50] and light scattering. The results indicated that all of the 23 mutants were monomeric at studied concentrations except for E154V, which appeared aggregation-prone. We excluded E154V from the subsequent analysis.

As shown in S2 Table, all single mutants are catalytically active except for D27F. D27 is known to be a key catalytic residue of *E*. *coli* DHFR [51].

For each mutant we obtained two measures of stability: the apparent melting temperature determined by Differential Scanning Calorimetry (DSC) and the urea midpoint unfolding concentration (*C*_m_) determined by monitoring chemical denaturation by Circular Dichroism (CD) with subsequent fitting to a two-state model (see Materials and Methods). Both measures of stability were highly correlated, despite the fact that thermal unfolding was irreversible (S7 Fig). Of the selected 22 single point mutations, 10 mutations were stabilizing, according to their *T*_m_ or *C*_m_ values (S2 Table). Given that statistically most random mutations are destabilizing with only a small fraction (less than 18%) stabilizing [8,12], this statistically significant result (*p* = 0.002 under the null hypothesis that mutations are random) indicates that MCPU is an effective method for selecting stability-enhancing mutations.

As expected, combinations of single stabilizing mutations led to more stable multiple mutant variants, [24,25,52] as predicted by simulation. In particular, the stability of the quintuple mutant (T68N,Q108D,T113V,E120P,S138Y) was found to be substantially higher than that of the wild type protein (Table 1), with *T*_m_ 7.2°C higher than WT, and *C*_m_, the urea concentration at the mid-unfolding point, was 0.43M higher than WT. All multiple mutants were catalytically active, and the quintuple mutant and triple mutant (T113V,E120P,S138Y) were found to be more catalytically active than WT. We note that while combination of stabilizing mutations generally increases stability, the effect is less than additive (S8 Fig); for instance, the quintuple mutant is about 4°C less stable than predicted under the assumption of additive **Δ***T*_m_ (a 7.2°C stability increase vs. predicted 9.6°C).

**Fig 7:**
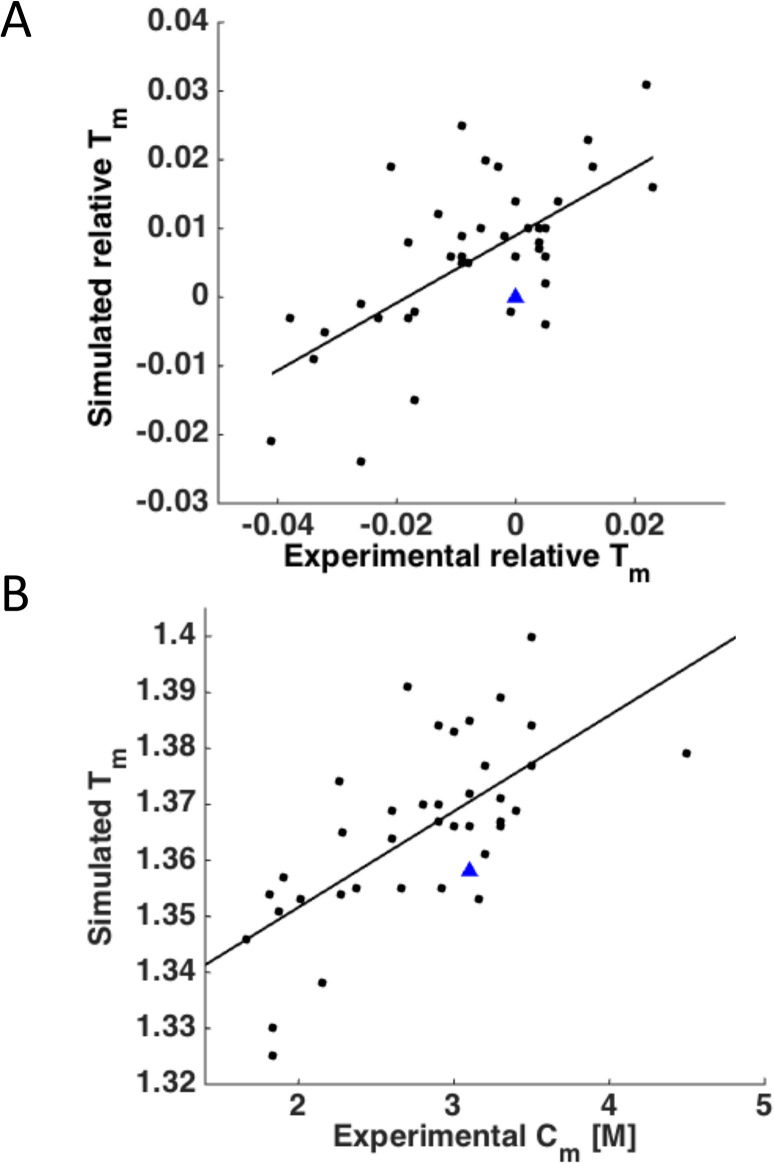
Correlation between the relative simulated and experimental *T*_m_ values. (A) Plot of simulated *T*_m_ vs. experimental *T*_m_. The relative *T*_m_ values were calculated by normalizing to WT: (*T*_m_(mutant)-*T*_m_(wild type))/ *T*_m_(wild type). Experimental values from this study and from Bershtein *et al*. [48] are included. WT is shown as a blue triangle. *r* = 0.65, *p* = 3 x 10^−6^. (B) Plot of simulated *T*_m_ vs. experimental C_m_. *r* = 0.68. *p* = 6 x 10^−7^.

We computationally predicted relative unfolding temperatures of 15 DHFR mutants published earlier [48] and added these mutants to the set for analysis resulting in 42 mutants in total. The correlation coefficient between experimental relative *T*_m_ and simulated relative *T*_m_ for the 42 mutants was about 0.65, as shown in Fig. 7A. To address the issue that both simulated *T*_m_ and DSC measurements are not strictly at equilibrium, we plotted the relation between simulated *T*_m_ and equilibrium measurement of stability in chemical denaturation by urea. The denaturation mid-transition urea concentration *C*_m_ and computationally determined unfolding temperature exhibit even a slightly higher correlation of *r* = 0.68 (Fig. 7B), demonstrating that our non-equilibrium simulation method shows good agreement with the equilibrium measurement of urea denaturation, as predicted by Equation 6.

**Fig 8:**
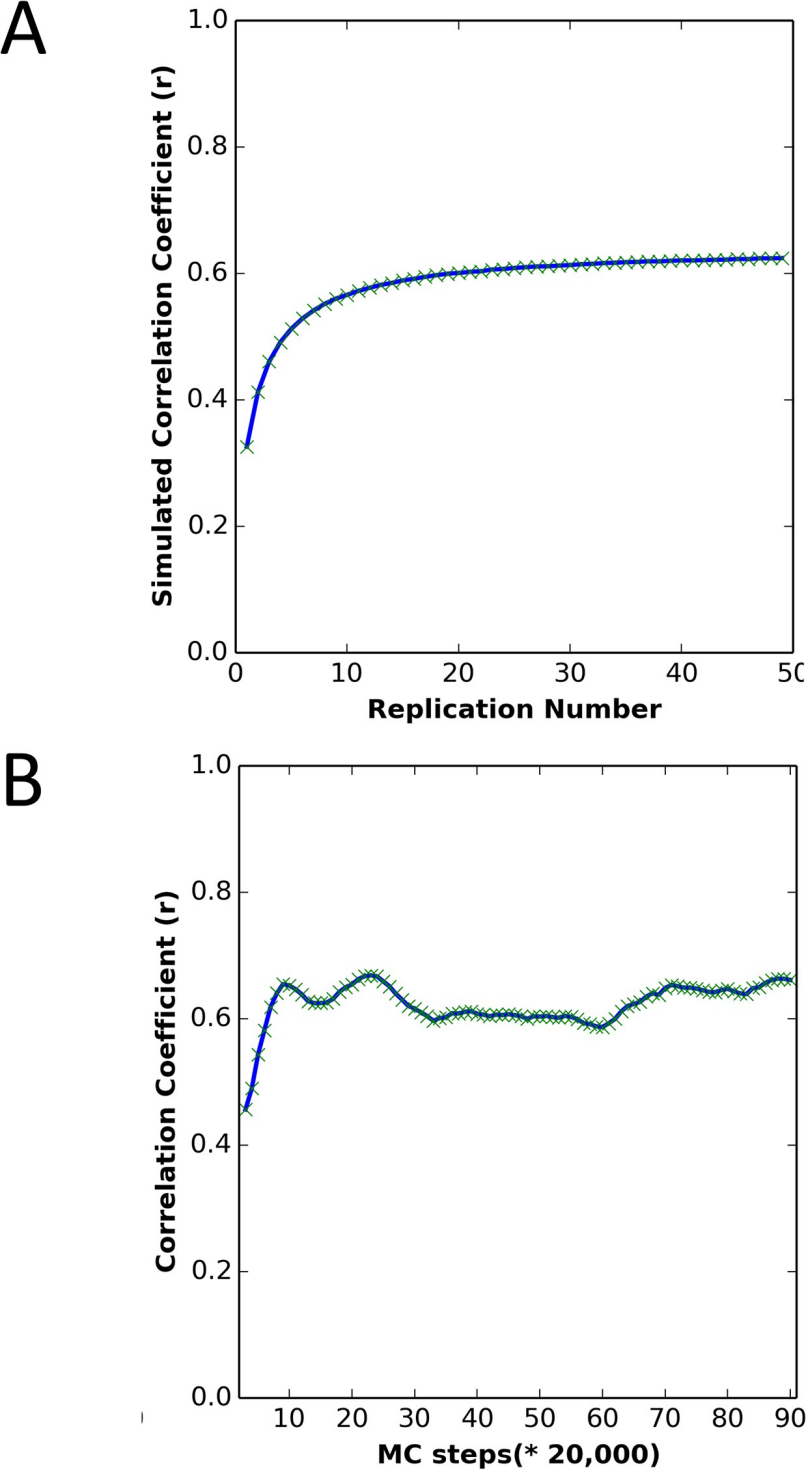
The effect of replication number and number of MC steps on simulation predictive power. (A) Correlation between simulated *T*_m_ and experimental *T*_m_, averaging over different numbers of replications, for the DHFR wild type and mutants. Each protein was simulated for 2,000,000 MC steps, following MD minimization and equilibration at low temperature. (B) Correlation between the simulated *T*_m_ and experimental *T*_m_ with different numbers of MC steps and 50 replications, for the DHFR wild type and mutants. Each protein was first simulated for the number of steps given on the x-axis, and the next 100,000 steps were averaged in determining the simulated *T*_m_.

We also used the dataset to evaluate the effect of the number of replications and the number of MC steps on the performance of the method. As shown in Fig. 8A, the prediction accuracy is sensitive to the number of replications. To achieve reliable *T*_m_ predictions, at least 20 replications should be used. However, the number of MC steps did not greatly affect prediction accuracy, provided simulations were run for at least ~ 200,000 steps (see Fig. 8B). In the context of the theory developed in the earlier section: “Predicting the effects of mutations on protein stability from non-equilibrium unfolding simulations,” this initial equilibration period may allow time for equilibration within the native basin, after which simulation length does not appreciably affect the consistency of results with equilibrium stability measurements.

### Stability and activity do not trade-off for DHFR

**Fig 9:**
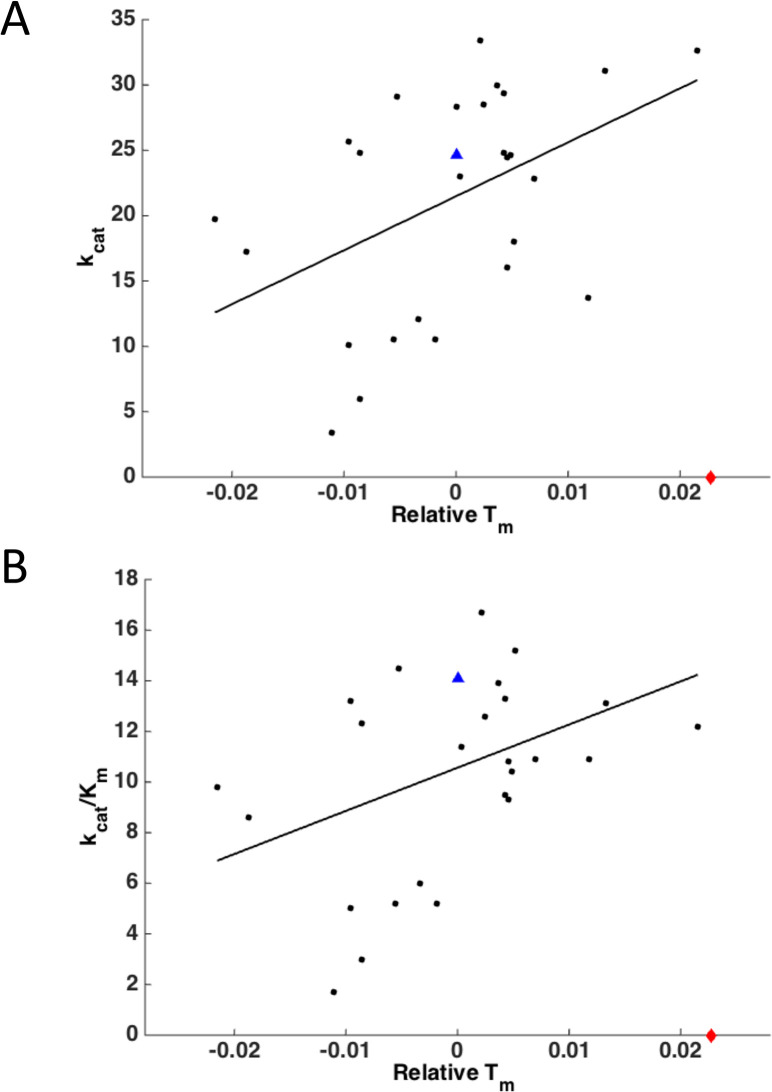
Correlation between DHFR activity and stability. WT is shown as a blue triangle; D27F is shown as a red diamond at zero activity. (A) Plot of *k*_cat_ vs. experimental relative *T*_m_. *r* = 0.46, *p* = 0.02 (excluding outlier D27F). (B) Plot of *k*_cat_/*K*_m_ vs. experimental relative *T*_m_. *r* = 0.41, *p* = 0.03 (excluding outlier D27F).

It has been proposed that stability imposes a constraint on protein function leading to stability-activity tradeoffs [53,54]. Our data, however, paints a different picture for DHFR—;of a weak *positive* correlation between *T*_m_ and *k*_cat_ or *k*_cat_/K_M_ (*r* = 0.46, *p* = 0.02 and *r* = 0.41, *p* = 0.03 respectively) with one notable outlier D27F, where the stabilizing mutation is made right in the active site (Fig. 9). The D27F mutant has high thermal stability but, as noted above, is not catalytically active, indicating that there is in fact a stability-activity trade-off for this active-site residue.

### Evolutionary analysis

Using an alignment of 290 bacterial DHFRs, we determined the DHFR consensus sequence (S9 Fig). Mutation of a non-consensus residue to the consensus residue generally resulted in protein stabilization [29]. In 4/16 of the experimentally stabilizing mutations, a residue was changed to the consensus residue, while only 2/29 destabilizing mutations resulted from a change to consensus. Likewise, in 18/29 destabilizing mutations, a residue was changed away from the consensus residue, while this was true for only 5/16 of stabilizing mutations.

### Simulated melting temperatures by residue

**Fig 10:**
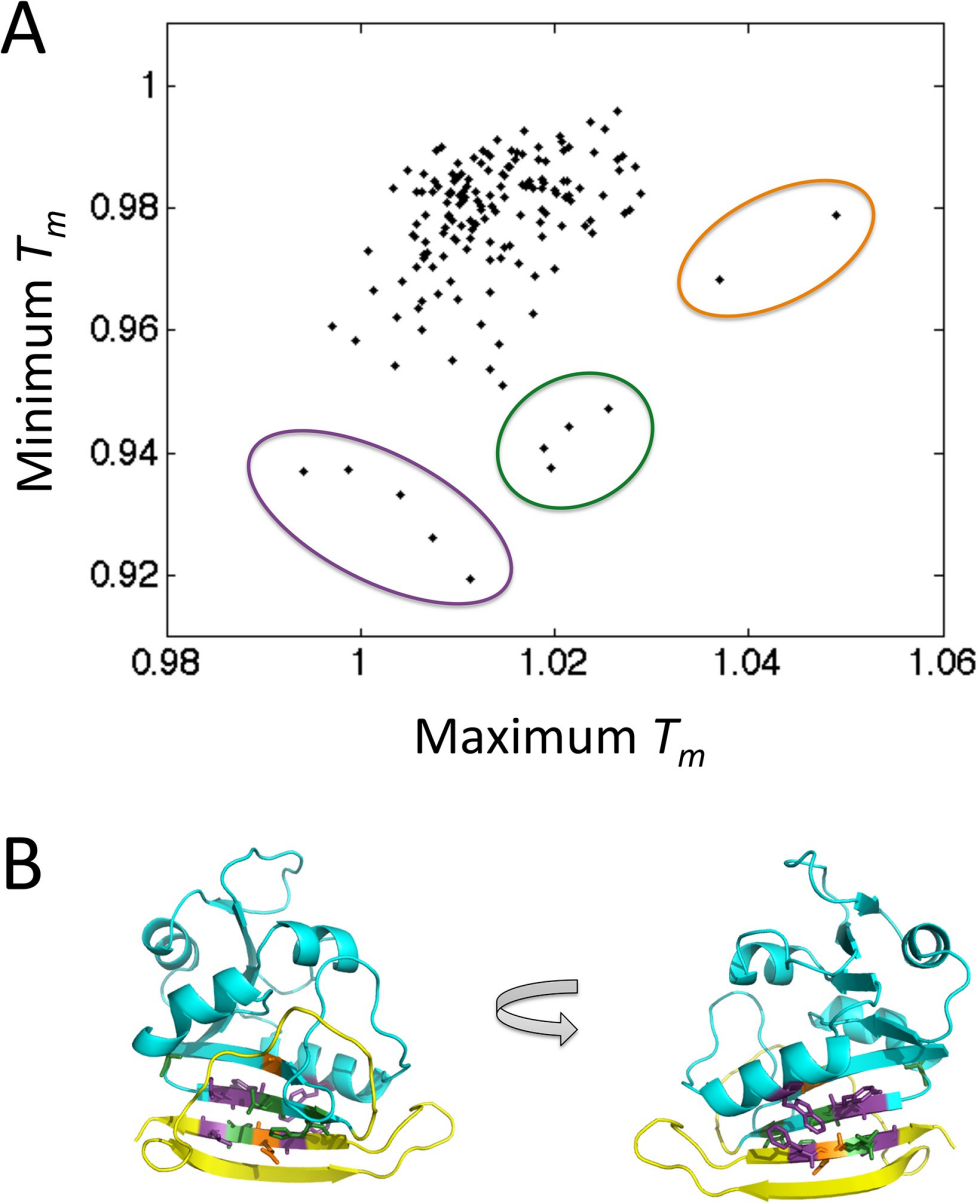
Maximum stabilization and destabilization induced by mutations at each residue position. (A) Plot comparing the minimum and maximum simulated *T*_m_ values, for each residue across all 19 simulated mutants. *T*_m_ is normalized to WT, by dividing each *T*_m_ value by the simulated WT *T*_m_ = 1.489 simulation units. Outliers are circled in purple (left), green (middle) and orange (right). (B) DHFR with outlier residues colored according to the color scheme from (A). Purple: residues F153, W30, Y111, L156, L110. Green: residues A107, I155, L112, H114. Orange: residues A6, E154. Excluding outlier residues, the C-terminal beta hairpin is colored yellow, and the rest of the protein is colored cyan.

We compared the minimum and maximum simulated *T*_m_ values obtainable by mutating a single residue to any of the 19 other amino acids (Fig. 10A). There is a weak positive correlation between minimum and maximum melting temperatures (*r* = 0.30, *p* = 10^−4^). Apparently, protein loci where mutations can cause significant stabilization are statistically less susceptible to destabilizing mutations and vice versa, which may be expected: once a residue is already at its most stabilizing amino acid variant, the protein cannot be stabilized further by mutation. Distinct outliers correspond to the loci with the strongest stabilizing or destabilizing effects of mutations. Interestingly, these outliers, which may represent structural weak spots in DHFR, tend to fall on the interface connecting the C-terminal beta hairpin and the rest of the protein (Fig. 10B). This is in fact the interface that is the first to dissociate in the Monte Carlo simulations (see Fig. 2).

### Comparison with other methods

We compared our computational DHFR predictions with four popular approaches to predict the effect of a mutation on protein stability: FoldX [17], Eris [26], PopMusic [55], and SDM [56]. (S3 Table). The MCPU performs better than these methods on DHFR mutants. PopMusic shows also strong performance with highly statistically significant *r* = 0.55 between theory and experiment, however the limitation of this method is that it can consider only single point mutations. To further evaluate MCPU performance we tested it on four additional proteins from four different SCOP structural classes: the Cro repressor protein from bacteriophage lambda (PDB-ID 5CRO), the *B*. *Subtilis* major cold shock protein (1CSP), *E*. *coli* Thioredoxin (2TRX), and Gln-25 ribonuclease T1 from *Aspergillus oryzae* (1RN1). Our predictions were compared with Eris and SDM. We did not compare MCPU results with FoldX and PopMusic as these mutations were selected in the training dataset for the two methods. As shown in Table 2, the correlation coefficient between MCPU predictions and the experimental *T*_m_ values, averaged over all proteins, is about 0.71, which is higher than that provided by Eris (-0.05), for which predictions were quite poor for both DHFR and other proteins, and SDM (0.63). If we consider only the binary prediction of whether a mutation is stabilizing or destabilizing, MCPU can correctly predict 11 out of 16 mutations, while Eris and SDM correctly classify 9 and 8 mutations respectively.

**Table 2:** Simulation results on non-DHFR proteins.

SCOP	Length	PDB	Mutant	Real Tm	MCPU	Eris	SDM	Native energy
All alpha proteins	66	5CRO	Y26D	54.0	0.878	−6.958	−0.690	−2044.4
	66	5CRO	Y26H	49.5	0.869	−3.425	−0.660	−1908.2
	66	5CRO	Y26L	46.0	0.868	−3.458	0.300	−1891.8
	66	5CRO	WT-5CRO	39.5	0.869	0.000	0.000	−1886.0
	66	5CRO	Y26W	37.5	0.871	−1.200	0.350	−1872.7
All beta proteins	67	1CSP	A46E	48.6	1.015	1.203	0.020	−1624.9
	67	1CSP	E3L	62.7	1.033	2.271	−0.460	−1536.7
	67	1CSP	E3R	69.6	1.023	1.638	−0.650	−1878.0
	67	1CSP	E66L	66.4	1.042	2.510	1.320	−1568.1
	67	1CSP	WT-1CSP	53.6	1.022	0.000	0.000	−1596.3
Alpha and beta proteins (a/b)	109	2TRX	D26I	98.0	1.135	2.847	4.290	−2478.5
	109	2TRX	WT-2TRX	87.0	1.107	0.000	0.000	−2558.4
	109	2TRX	T66L	85.0	1.124	3.540	2.180	−2552.0
	109	2TRX	T77V	82.0	1.124	−0.980	1.960	−2541.9
	109	2TRX	C35A	73.0	1.104	−14.670	−2.040	−2574.7
Alpha and beta proteins (a+b)	125	1RN1	V16A	44.5	0.887	2.033	−1.530	−1934.1
	125	1RN1	V16S	36.9	0.876	3.027	−4.410	−1920.1
	125	1RN1	V78S	34.6	0.878	3.870	−4.450	−1943.8
	125	1RN1	V89S	29.6	0.877	3.414	−4.100	−1904.8
	125	1RN1	WT-1RN1	51.5	0.899	0.000	0.000	−1929.7
				Error number	5	7	8	7
				Error rate	0.313	0.438	0.500	0.438
				*r*	0.708	−0.053	0.635	−0.348

Error number and error rate describe the number and fraction of mutations not predicted in the correct direction (stabilizing vs. destabilizing)

### Entropy of the native state is an important contributor to stability

The theoretical analysis of the unfolding simulations relates the effect of mutations on the equilibrium between folded and unfolded states to the effect of mutations on free energy of the folded and transition states. It is widely believed that in the low-entropy folded state energetic factors dominate. If so that would imply that we can get an equally good correlation between prediction and experiment by estimating the mutational effect on energy of the native state as is the case for most empirical methods. To that end we evaluated the correlation between the energy of the minimized (after long MC equilibration) native state and the experimental *T*_m_ and found only a weak correlation with experimental melting temperatures (Table 2, last column), indicating that protein entropy, which is accounted for in the MCPU, in addition to enthalpy, is important in determining protein stability.

## Discussion

Estimates of protein stability using Molecular Dynamics are prohibitive for all but the smallest protein domains. However using MCPU we were able to efficiently explore stabilities of all possible point mutants for an essential enzyme of a typical size (159 amino acids) in a manageable amount of computational time (approx. one hour for every 1,000,000 MC steps). Although the use of rapid Monte Carlo simulations reduces simulation time and allows for a greater number of replicates, our method to predict stability effects of mutations based on non-equilibrium unfolding simulations represents a general approach that could be modified for use with conventional MD simulations, especially given the current rate of improvement in simulation speed and accuracy.

Since our method involves protein unfolding simulations and not equilibrium simulations of both folding and unfolding processes, we expect it to be especially useful for predicting mutations that mostly affect the rate of protein unfolding as highlighted in our theoretical analysis. Low *φ*-value residues, which acquire contacts with other residues late in the folding process and lose contacts early in the unfolding process [14] constitute the majority of residues in proteins, with *φ*-value roughly constant around 0.24 as noted in [45]. Combining this observation with assumptions of transition state theory, we found that for the majority of residues (those not part of the folding nucleus [14,57] exhibiting anomalously high *φ*-values) the observed simulation *T*_m_ relative to WT is proportional to the equilibrium stability change ΔΔ*G*, as verified by simulation and experiment. We establish that relative *T*_m_ is independent of simulation length, demonstrating that non-equilibrium simulations can in fact be used to quantify relative protein stability.

Many of the experimentally verified stabilizing mutations in DHFR predicted by MCPU are found in the C-terminal beta hairpin region, which is the first to unfold in simulations, prior to the main unfolding event encompassing the entire structure (see Fig. 2). It has been shown that the source of ultra-stability in hyperthermophiles generally arises from slowing the unfolding rate, rather than increasing the folding rate [28], so our method may be particularly suitable for discovering biologically relevant stabilizing mutations. In addition, our results might be particularly applicable to *in vivo* studies, where protease digestion and/or aggregation proceed from the partially-unfolded state. We note, however, that some stabilizing residues predicted by MCPU lie in the region of the protein that is late to unfold in simulations, including I61V, which raises the experimental melting temperature by 1.7°C. These mutants, along with the destabilized outlier I155A for which relative *T*_m_ depends on simulation length (Fig. 6), are appealing candidates for further study, as they may reflect a breakdown in the simplifying assumptions of 2-state kinetic theory for proteins.

It has been hypothesized that there exists a tradeoff between enzyme activity and stability, since certain regions of an enzyme must be sufficiently flexible to promote catalysis [53,54]. This conclusion was reached in [53,58], based on the exploration of stability effects of mutations in the active site of beta-lactamase [53] and rubisco [58]. Fersht and coauthors also found several stabilizing mutations in the active site of Barnase rendering the protein inactive [59]. While we observe a similar effect with the D27F mutation in DHFR, Fig. 9 shows that exploring only mutations in the active site provides a biased view on the tradeoff between activity and stability. Rather a vast majority of mutations throughout the protein show a qualitatively opposite trend. The likely explanation of the distinction between an apparent tradeoff when mutations are made in the active site and the opposite trend for mutations outside of the active site is that “carving” an active site requires special selection of catalytic amino acids, which could indeed have a destabilizing effect, overall. However our observation of a small positive correlation argues against an obligate relation between global protein dynamics and activity for DHFR, at least for the aspects of dynamics that are correlated with stability. Warshel and colleagues reached a similar conclusion in their theoretical analysis of the role of dynamics for DHFR and other proteins in [60]. This point has likewise been made by Bloom *et al*. [11], who noted that a number of proteins have been stabilized experimentally without loss of activity, and Taverna and Goldstein argued that marginal stability is an inherent property of proteins due to the high dimensionality of sequence space and not due to a requirement of reduced stability in order to generate sufficient flexibility [61].

A straightforward explanation for the weak yet statistically significant *positive* correlation between activity and stability observed in our case might be that more stable proteins have greater effective concentration of the folded (*i*.*e*. active) form. It is also important to note that a weak yet statistically significant positive correlation between activity and stability for DHFR can be revealed only when stabilizing mutations are included in the analysis. Our earlier study [48] analyzed a smaller set of primarily destabilizing mutants and did not reveal any statistically significant trend (positive or negative) in the stability-activity relation for DHFR.

The development of highly-stabilized DHFR mutants through our combined *in silico—;in vitro* approach opens up promising avenues for new *in vivo* studies. It has been postulated that protein stability places a fundamental constraint on the evolutionary pathways available to a protein [29,62] which has particular significance in the development of antibiotic resistance: higher protein stability can provide the microorganism with an increased capacity to evolve to evade antibiotic drugs [63] or, more generally, with capacity to evolve new functions [62]. We plan to use an approach developed in our lab [48] to endogenously introduce stabilized DHFR mutants into the bacterial chromosome and we will evaluate mutant fitness relative to wild-type using growth rates and competition experiments. These experiments will allow us to assess whether an evolutionary trade-off exists between stability and fitness *in vivo*, particularly in the presence of antibiotics.

We plan to apply MCPU to predict stability effects of mutations in proteins other than DHFR, in particular to develop highly stabilized mutants. Comprehensive experimental analysis of fitness and/or stability effects of mutations [64] could be useful in assessing the predictive capabilities of this method. In addition to predicting mutant stabilities, MCPU can provide atomic-detail molecular trajectories to rationalize the stability effects of mutations; such analysis is left to future study.

## Materials and Methods

### Monte Carlo simulations

We employed an all-atom Monte Carlo simulation program incorporating a knowledge-based potential, described in previous publications [16,31,65]. Briefly, the energy function is a sum of contact energy, hydrogen-bonding, torsional angle, and sidechain torsional terms, with an additional term describing orientation of nearby aromatic residues. The move set consists of rotations about ϕ, ψ, and χ dihedral angles, with bonds and angles held fixed. Moves are accepted or rejected according to the Metropolis criterion.

Mutations were introduced into the protein using the program Modeller v9.2 [66]. An initial minimization was carried out in NAMD [67] for 5,000 steps, using the default minimization algorithm and par_all27_prot_lipid.inp parameter file (without waters). An additional minimization step was carried out by running the Monte Carlo simulation program at low temperature (0.100 in simulation units) for 2,000,000 steps. A 2,000,000-step simulation was then run at each of 32 temperatures, averaging over all 2,000,000 steps to obtain Energy, RMSD, and number of contacts. These results were averaged over 50 simulations, for each temperature. Data was then plotted and fit to a sigmoid to obtain the computationally-predicted melting temperature, for each of Energy, RMSD, and number of contacts. To assess dependence of melting temperature on simulation length, longer simulations of 20,000,000 steps were carried out with 30 replications, averaging over the final 2,000,000 steps. For DHFR, 1,000,000 steps took approximately one hour of simulation time, on a single CPU.

#### Bioinformatics Analysis

DHFR protein sequences from 290 bacterial species were aligned using the program MUSCLE and online server [68]. MATLAB, with the Bioinformatics Toolbox, was used to create sequence logo representations and to determine the consensus sequence.

### Effect of number of replications on simulation accuracy

We evaluated the effect of the number of MC simulation replications on the prediction results. As shown in S9 Fig, the prediction accuracy is sensitive to the number of replications, but converges to a constant value after approximately 20 replications. In addition, we saw that increasing the number of MC steps beyond 2,000,000 steps does not increase prediction accuracy when the protein has been simulated with at least 20 replications, despite the fact that not all simulations have converged by 2,000,000 steps (S2 Fig—;S4 Fig).

### Simulation analysis

Sigmoidal fits were accomplished using the module “Sigmoidal, 4PL” using the software program Prism 6. The sigmoid function has the form:

Y = Bottom + (Top-Bottom)/(1+10^((LogIC50-X)*HillSlope))

### Method availability

The tool is accessible from Shakhnovich lab website http://faculty.chemistry.harvard.edu/shakhnovich/software

### Site-directed protein mutagenesis of DHFR

The wild type dhfr gene was cloned in a pET24 expression vector under the inducible T7 promoter, then transformed into BL21(DE3) cells [69]. Single point mutations of DHFR were constructed based on a two-step PCR-mutagenesis strategy [70], in which the template for the PCR is the plasmid of WT DHFR. The multiple-mutant variants of DHFR were constructed based on the same method with the single point mutation, but the template of PCR was the plasmid of the corresponding dhfr mutant. To verify the mutations of dhfr, DNA sequencing was performed at the GENEWIZ Incorporation (MA, U.S.). The verified plasmids were transformed into competent *E*. *coli* BL21(DE3) cells for expression.

### Protein expression and purification

WT DHFR and all mutants used in this study were cloned into a pET24 expression vector and overexpressed in the BL21(DE3) pLys *E*. *coli* strain.

A single colony of the transformed *E*. *coli* carrying the wild type or mutation dhfr was cultured in Luria-Bertani liquid medium containing 50 μg/mL kanamycin (LB-kana) at 30°C overnight, and then inoculated to fresh LB-kana (1:100 dilution) and incubated again at 30°C. When the OD_600_ of the culture reached 0.6, isopropyl β-D-1-thiogalactopyranoside (final concentration, 0.4 mM) was added. Cultures were incubated for an additional 12–16 h at 25°C. The cells were then collected by centrifugation and disrupted by sonication. The recombinant proteins were purified with Ni-NTA Superflow (QIAGEN, U.S.) according to the manufacturer’s instructions. Then, the collected protein sample was run with Superdex 75pg Column and was desalted with the desalting Column in ÄKTA protein purification system (GE Healthcare, U.S.). The final concentration of the purified protein was determined using the BCA protein assay kit (PIERCE CHEMICAL, USA) or the NanoDrop instrument (GE Healthcare, U.S.).

### Enzyme kinetics

DHFR kinetic parameters were measured by the progress-curve kinetics, essentially as described [69,71]. A Scientific stopped flow apparatus, RX.2000 Rapid kinetics system (Applied Photophysics, UK) was used with absorbance monitoring at 340 nm, under single-turnover conditions. NADPH was preincubated with DHFR for 5 min in syringe 1 at the temperature 25°C in a thermostated syringe compartment, and then the reaction was initiated by rapidly mixing the contents with dihydropholate (DHF) from syringe 2. The final assay conditions are 25 nM DHFR, 120 μM NADPH(D), and 25 μM DHF in MTEN buffer (50 mM 2-(N-morpholino)ethanesulfonic acid, 25 mM tris(hydroxymethyl)aminomethane, 25 mM ethanolamine, and 100 mM sodium chloride, pH 7.6). The kinetics parameters (*k*_cat_ and *K*_M_) were derived from progress-curves analysis using Global Kinetic explorer [72].

### Stability measurements

Thermal stability was characterized by differential scanning calorimetry (DSC), essentially as described in references [69,73]. Briefly, DHFR proteins in Buffer A (10 mM potassium-phosphate buffer pH 7.8 supplemented with 0.2 mM EDTA and 1 mM beta-mercaptoethanol) were subjected to a temperature increase of 1°C/min between 20 to 90°C (nano-DSC, TA instruments, U.S.), and the evolution of heat was recorded as a differential power between reference (buffer A) and sample (120 μM protein in buffer A) cells. The resulting thermogram (after buffer subtraction) was used to derive apparent thermal transition midpoints (Tm app). Thermal unfolding appeared irreversible for all DHFR proteins tested [48], and the two state scaled model provided in NanoAnalyze software (TA INstruments, U.S.) was used to fit the Tm app value. The mutants constructed in this study and the ones published earlier [48] were determined with different DSC instruments with slightly different calibration leading to a small offset of about 2°C for the WT DHFR for earlier published data[48].

Urea unfolding was used to measure stability of the DHFR mutants against chemical denaturation. Proteins (25 μM in buffer A) were diluted in urea (0.2 mM increments up to a final urea concentration between 0 and 6 M), preequilibrated overnight at 25°C for 3 hours, and the change in the folded fraction was monitored by a circular dichroism signal at far-uv wavelength (221 nm) at 25°C (J-710 spectropolarimeter, Jasco). Fitting to a two-state model was used to derive the chemical transition midpoint (*C*_m_).

## Supporting Information

S1 Fig The MCPU flow chart. (TIF)

S2 Fig The RMSDs of DHFR wild type and mutants I115A and I155T vs. Monte Carlo step at temperatures from 0.1 to 3.2. RMSD is averaged over 50 replications. (TIFF)

S3 Fig The total energy of DHFR wild type and mutants I115A and I155T vs. Monte Carlo step at temperatures from 0.1 to 3.2. Total energies are averaged over 50 replications.
(TIFF)

S4 Fig The number of contacts for DHFR wild type and mutants I115A and I155T vs. Monte Carlo step at temperatures from 0.1 to 3.2. Number of contacts were averaged over 50 replications.
(TIFF)

S5 Fig The unfolding curves of DHFR and mutants I115A and I155T. Data points are averaged over the 2,000,000 step simulation and 50 separate runs.
(TIFF)

S6 Fig Energy vs. Temperature simulated melting curve for WT DHFR and mutants I115A and I155T, averaging over the last 2,000,000 steps of a 20,000,000- step simulation, and 30 separate runs. The mutant melting temperatures show the same trend as in S5 Fig, although all melting temperatures are shifted to lower values (since the protein is given more time to unfold at each temperature), and the curve deviates more notably from a sigmoid. (A) Overlaid data points from all three mutants. (B) Fits to a sigmoidal function (blue line), for each of the three mutants.
(TIFF)

S7 Fig Correlation between urea denaturation midpoint, *C*_m_, and melting temperature *T*_m_, for WT and 22 mutants predicted to be stabilizing by MCPU. r = 0.81, p = 2 x 10^−6^. Blue triangle denotes WT, red diamond denotes D27F.
(TIF)

S8 Fig Protein stabilization for multiple mutants: deviation from additivity. Change in experimental melting temperature relative to WT is predicted by summing melting temperature changes of individual mutants. This predicted **Δ***T*_m_ is plotted relative to the observed **Δ***T*_m_ (blue circles). r = 0.80, p = 0.06. Red line denotes predicted **Δ***T*_m_ = observed **Δ***T*m.
(TIFF)

S9 Fig Sequence alignment and sequence entropy for 290 bacterial DHFRs. Sequence Logo for the alignment, generated using the MATLAB Bioinformatics Toolbox.
(TIF)

S1 Table The simulated Tm values of the selected single point mutations and WT DHFR. (DOCX)

S2 Table The experimental results for all single point mutations of DHFR. (DOCX)

S3 Table Stability predictions for DHFR mutants, using MCPU/other methods. (DOCX)
