# Supplementary figures and images for: Thermal Stabilization of Dihydrofolate Reductase Using Monte Carlo Unfolding Simulations and Its Functional Consequences
Source: PLoS Comput Biol. 2015 Apr 23;11(4):e1004207. doi: 10.1371/journal.pcbi.1004207 (PMC4407897; doi:10.1371/journal.pcbi.1004207)

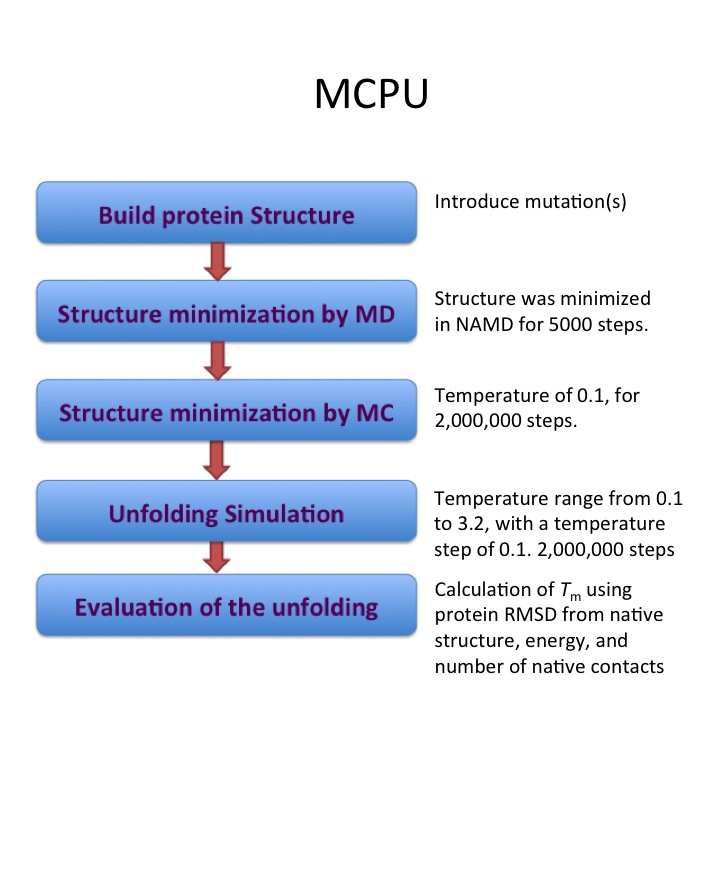

Supplement: S1 Fig — (TIF) [file pcbi.1004207.s001.tif]

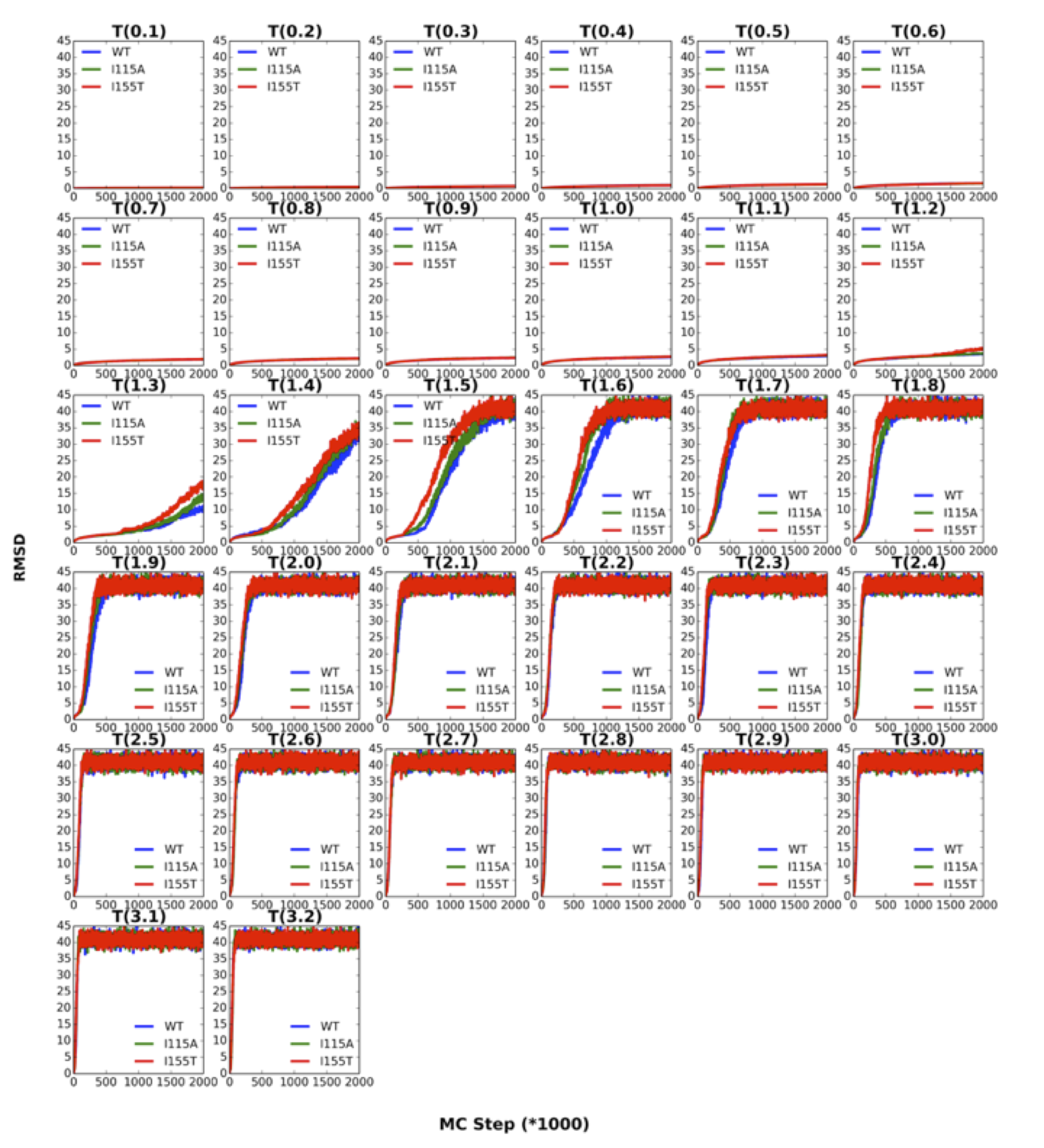

Supplement: S2 Fig — (TIFF) [file pcbi.1004207.s002.tiff]

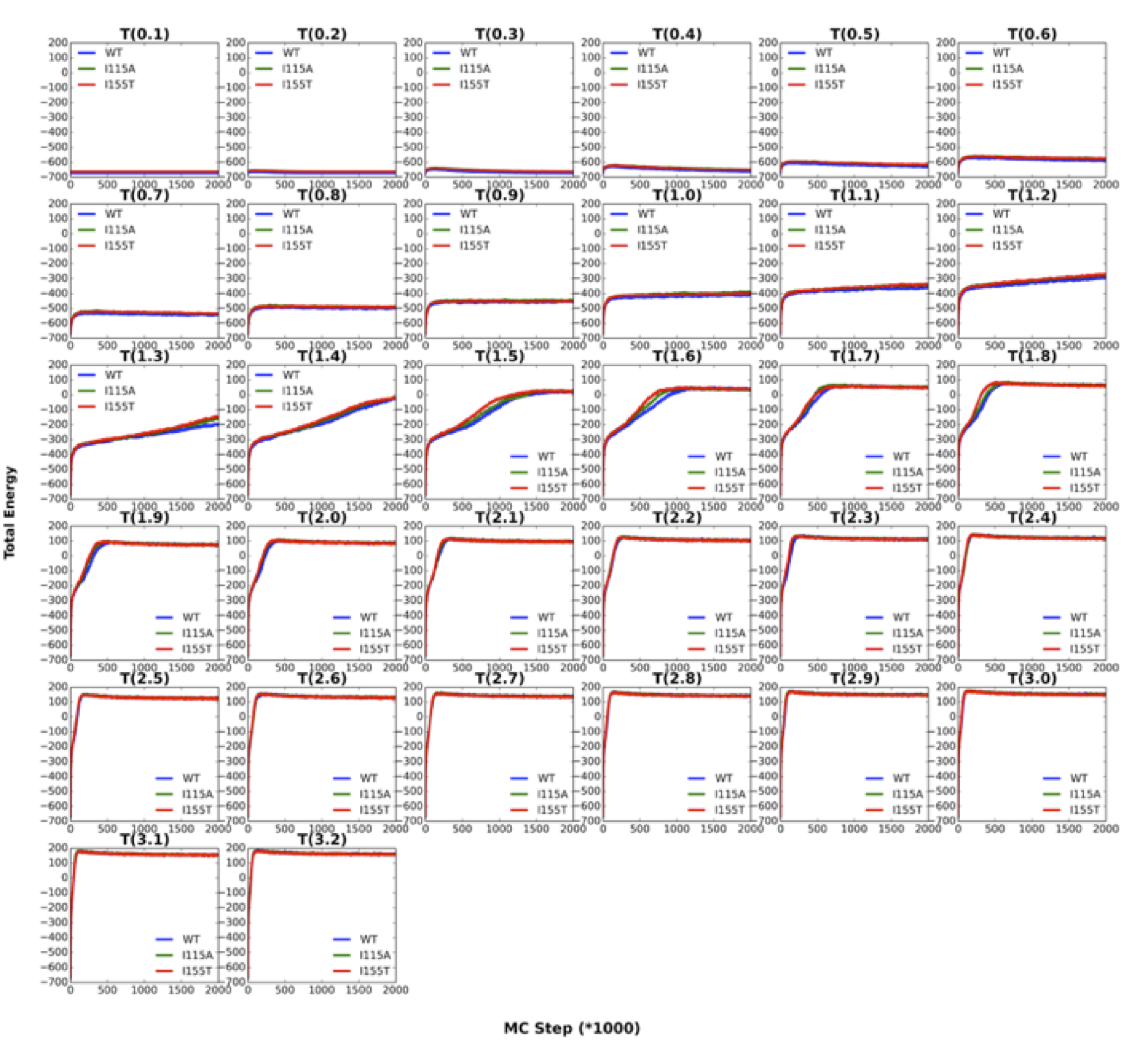

Supplement: S3 Fig — Total energies are averaged over 50 replications. (TIFF) [file pcbi.1004207.s003.tiff]

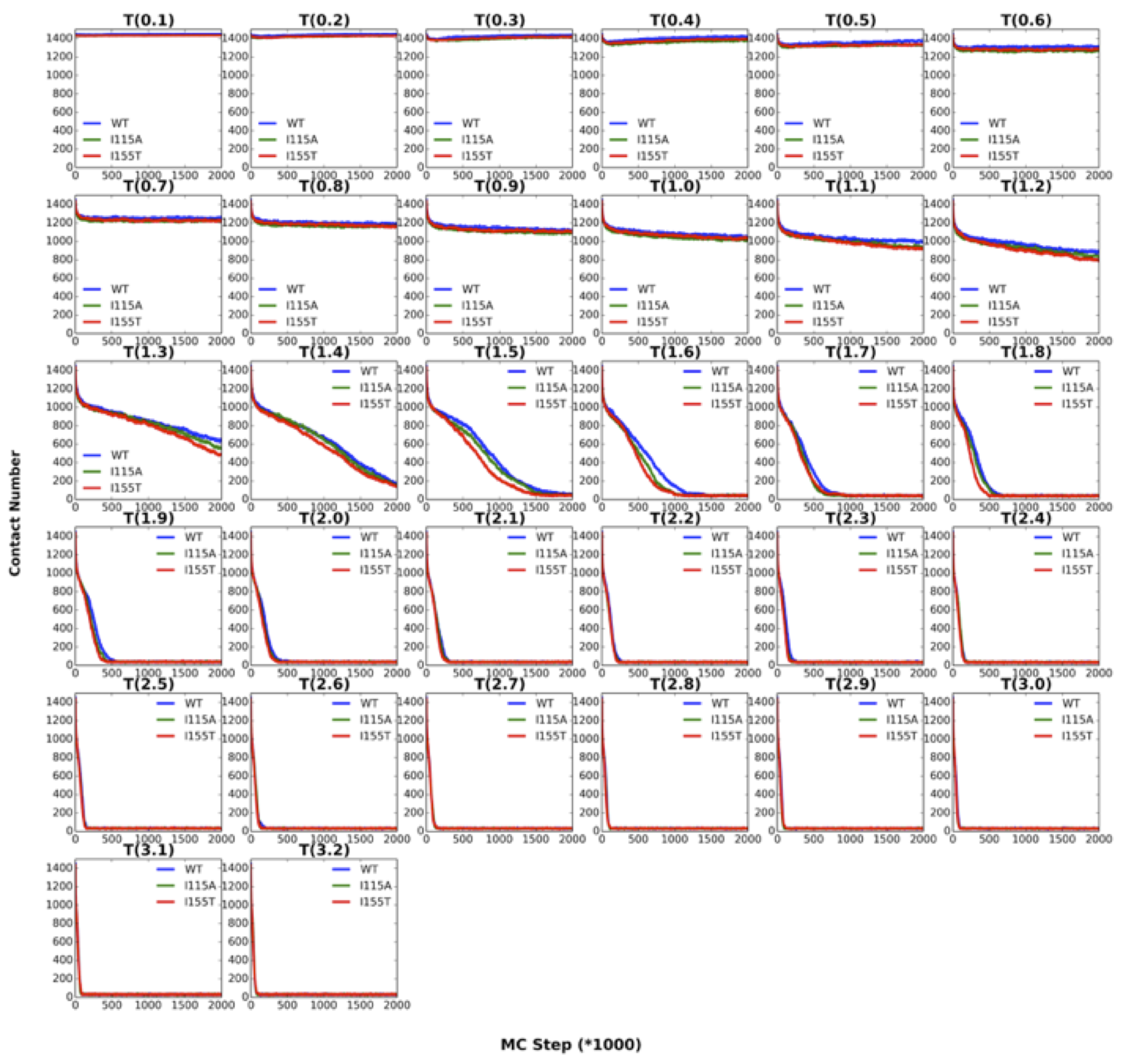

Supplement: S4 Fig — Number of contacts were averaged over 50 replications. (TIFF) [file pcbi.1004207.s004.tiff]

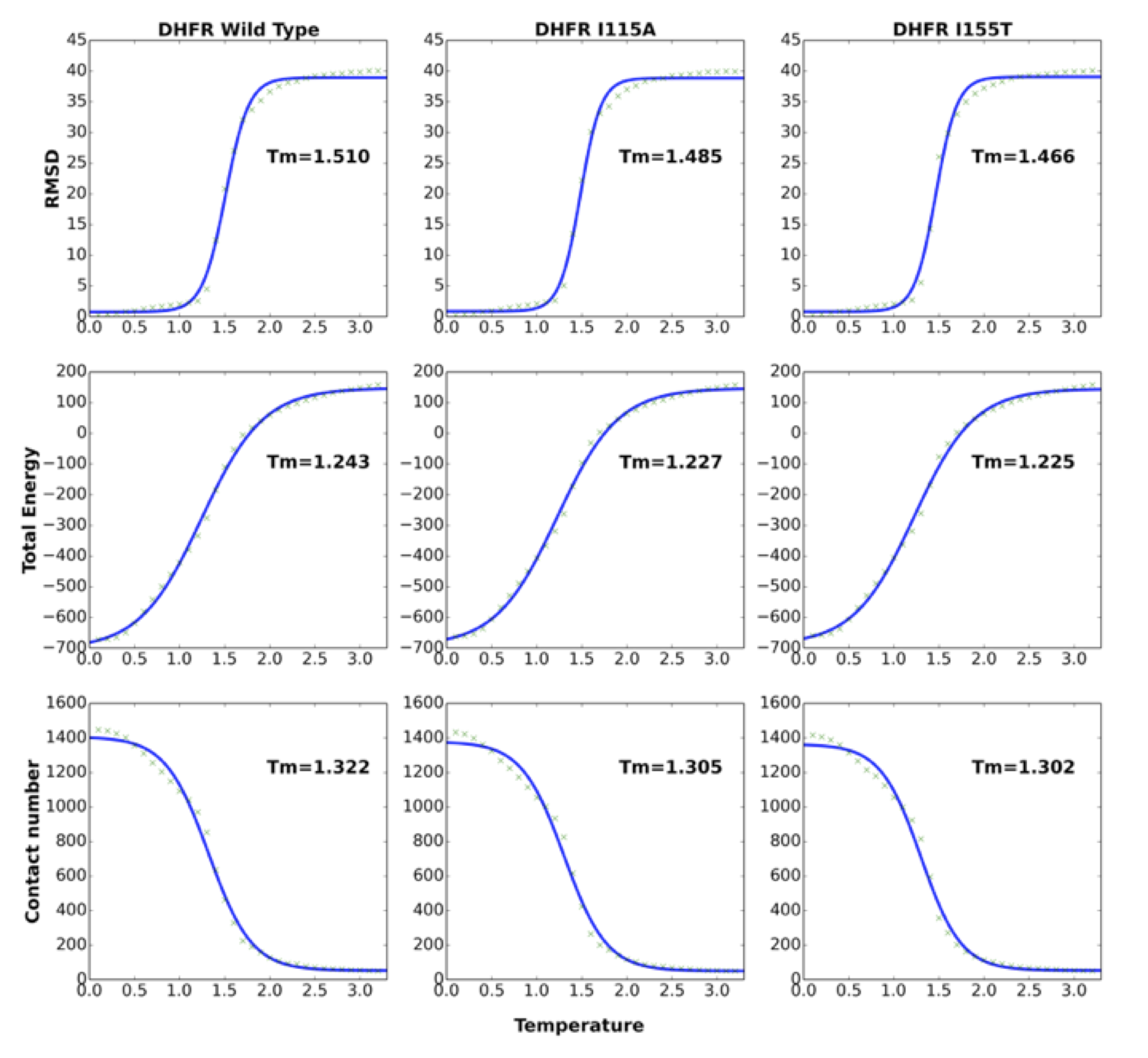

Supplement: S5 Fig — Data points are averaged over the 2,000,000 step simulation and 50 separate runs. (TIFF) [file pcbi.1004207.s005.tiff]

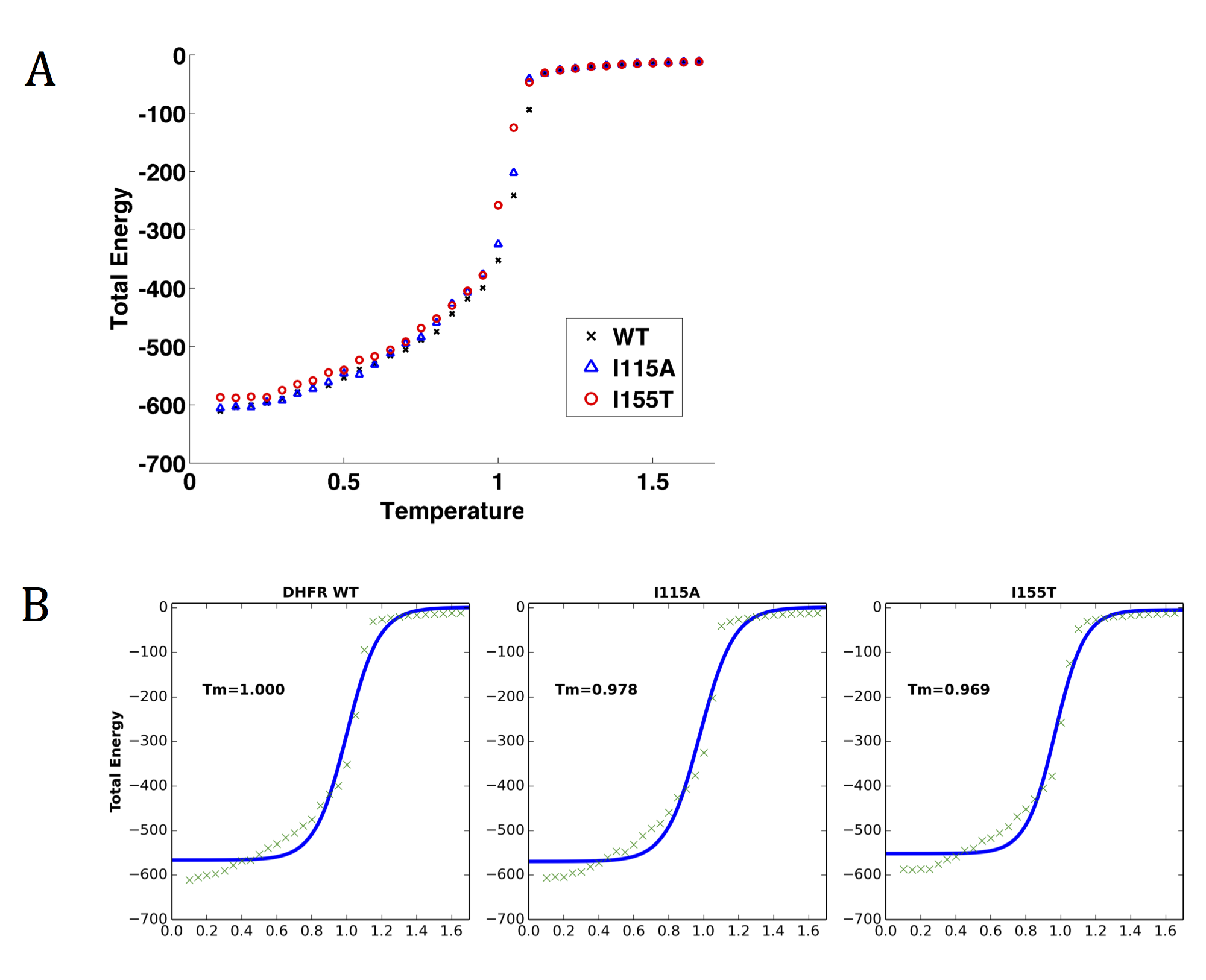

Supplement: S6 Fig — The mutant melting temperatures show the same trend as in S5 Fig, although all melting temperatures are shifted to lower values (since the protein is given more time to unfold at each temperature), and the curve deviates more notably from a sigmoid. (A) Overlaid data points from all three mutants. (B) Fits to a sigmoidal function (blue line), for each of the three mutants. (TIFF) [file pcbi.1004207.s006.tiff]

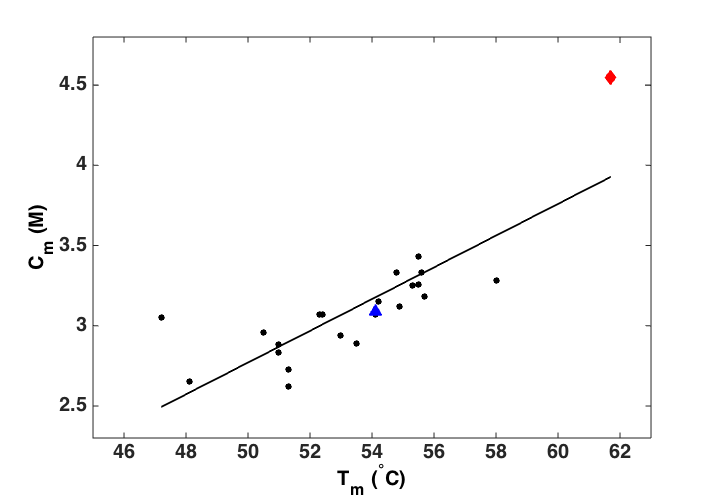

Supplement: S7 Fig — r = 0.81, p = 2 x 10^−6^. Blue triangle denotes WT, red diamond denotes D27F. (TIF) [file pcbi.1004207.s007.tif]

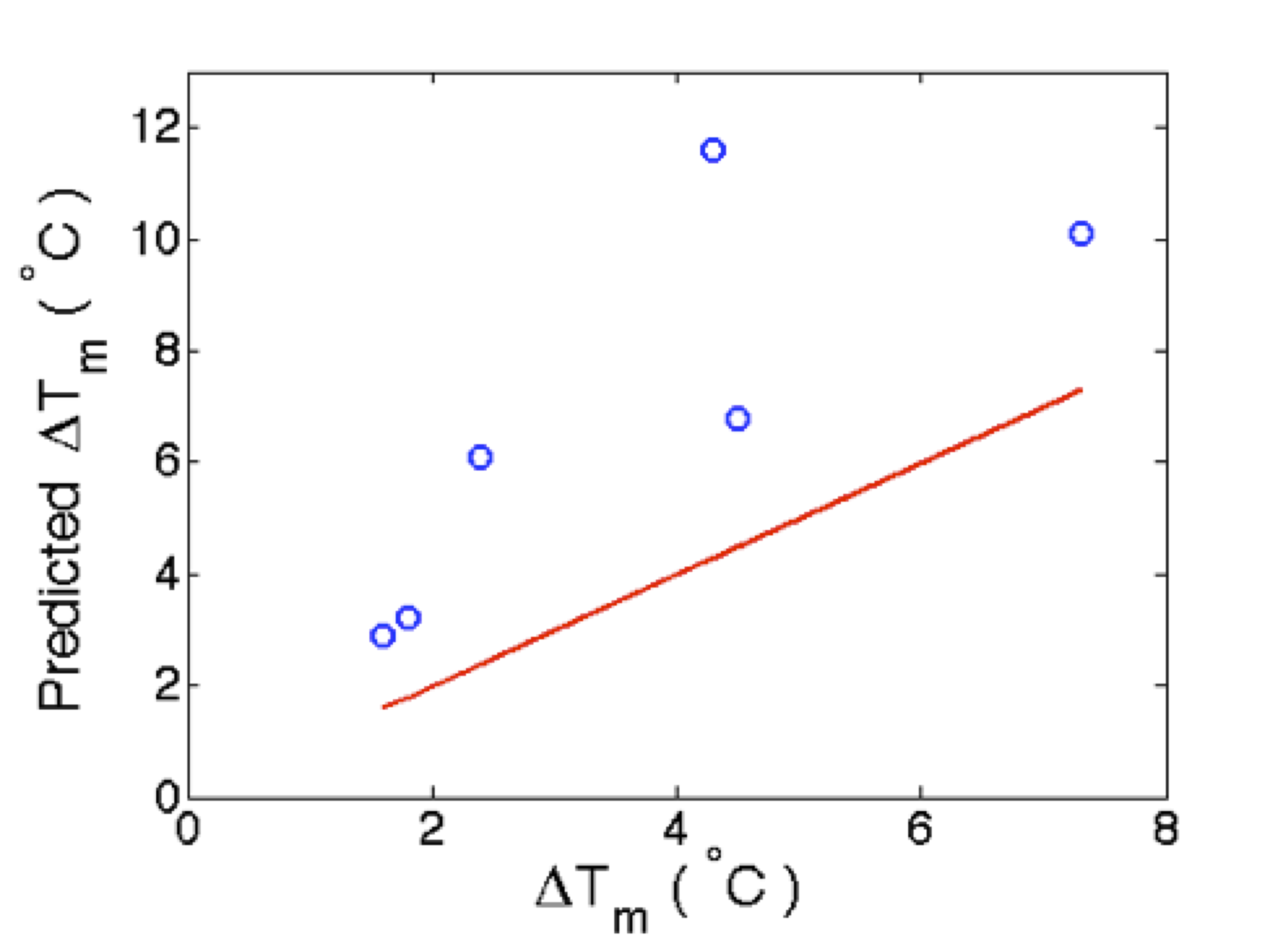

Supplement: S8 Fig — Change in experimental melting temperature relative to WT is predicted by summing melting temperature changes of individual mutants. This predicted ΔTm is plotted relative to the observed ΔTm (blue circles). r = 0.80, p = 0.06. Red line denotes predicted ΔTm = observed ΔTm. (TIFF) [file pcbi.1004207.s008.tiff]

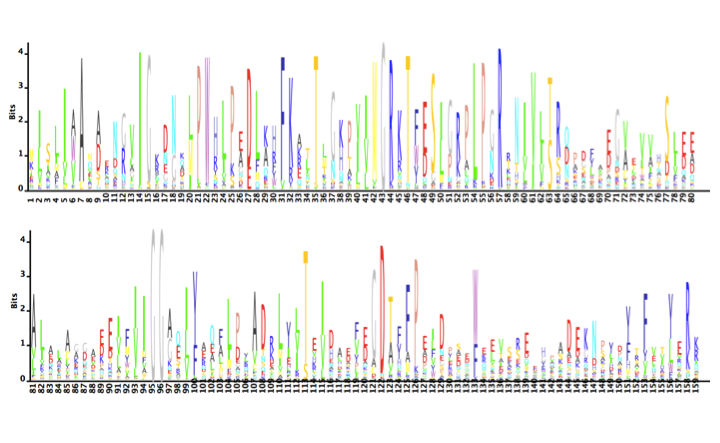

Supplement: S9 Fig — Sequence Logo for the alignment, generated using the MATLAB Bioinformatics Toolbox. (TIF) [file pcbi.1004207.s009.tif]
