# Supplementary material for: Thermal Stabilization of Dihydrofolate Reductase Using Monte Carlo Unfolding Simulations and Its Functional Consequences
Source: PLoS Comput Biol. 2015 Apr 23;11(4):e1004207. doi: 10.1371/journal.pcbi.1004207 (PMC4407897; doi:10.1371/journal.pcbi.1004207)
Supplement: S1 Table — (DOCX) [file pcbi.1004207.s010.docx]

| Mutations | *T*_m_  (RMSD) | *T*_m_  (Total Energy) | *T*_m_  (Contact Number) | Average *T*_m_ |
| --- | --- | --- | --- | --- |
| WT | 1.507 | 1.243 | 1.323 | 1.358 |
| D27F | 1.525 | 1.261 | 1.351 | 1.379 |
| T113V | 1.551 | 1.259 | 1.358 | 1.389 |
| Q108D | 1.510 | 1.244 | 1.329 | 1.361 |
| S138Y | 1.518 | 1.247 | 1.333 | 1.366 |
| D116F | 1.525 | 1.248 | 1.334 | 1.369 |
| T68N | 1.516 | 1.250 | 1.336 | 1.367 |
| E120P | 1.519 | 1.257 | 1.337 | 1.371 |
| V119F | 1.519 | 1.252 | 1.344 | 1.372 |
| S135I | 1.527 | 1.248 | 1.340 | 1.371 |
| C152I | 1.534 | 1.253 | 1.345 | 1.377 |
| H114R | 1.506 | 1.249 | 1.342 | 1.366 |
| S49E | 1.509 | 1.260 | 1.340 | 1.370 |
| H141F | 1.536 | 1.264 | 1.351 | 1.384 |
| E157F | 1.536 | 1.268 | 1.352 | 1.385 |
| G15W | 1.513 | 1.261 | 1.342 | 1.372 |
| E154V | 1.607 | 1.273 | 1.372 | 1.417 |
| L156Y | 1.510 | 1.247 | 1.334 | 1.364 |
| E139V | 1.548 | 1.271 | 1.355 | 1.391 |
| D87P | 1.510 | 1.251 | 1.339 | 1.367 |
| G43P | 1.510 | 1.263 | 1.336 | 1.370 |
| W74F | 1.512 | 1.252 | 1.334 | 1.366 |
| G67H | 1.515 | 1.254 | 1.339 | 1.369 |
| A6I | 1.542 | 1.260 | 1.347 | 1.383 |

Note: The data were simulated with 50 replications, for a total of 2,000,000 MC steps.
