# Supplementary material for: Thermal Stabilization of Dihydrofolate Reductase Using Monte Carlo Unfolding Simulations and Its Functional Consequences
Source: PLoS Comput Biol. 2015 Apr 23;11(4):e1004207. doi: 10.1371/journal.pcbi.1004207 (PMC4407897; doi:10.1371/journal.pcbi.1004207)
Supplement: S2 Table — (DOCX) [file pcbi.1004207.s011.docx]

| Mutation | *T*_m_(DSC) | *C*_m_(CD) | *k*_cat_ | *k*_cat_/*K*_m_ |
| --- | --- | --- | --- | --- |
| WT | 54.1 | 3.09 | 24.60 | 14.07 |
| D27F | 61.7 | 4.55 | N.D. | N.D. |
| T113V | 58.0 | 3.28 | 13.67 | 10.86 |
| Q108D | 55.7 | 3.18 | 24.60 | 10.35 |
| S138Y | 55.6 | 3.33 | 24.51 | 9.33 |
| D116F | 55.5 | 3.43 | 24.80 | 9.53 |
| T68N | 55.5 | 3.26 | 29.36 | 13.32 |
| E120P | 55.3 | 3.25 | 30.02 | 13.91 |
| V119F | 54.9 | 3.12 | 28.50 | 12.57 |
| S135I | 54.8 | 3.33 | 33.35 | 16.66 |
| C152I | 54.2 | 3.15 | 22.99 | 11.44 |
| H114R | 54.1 | 3.07 | 28.31 | 14.06 |
| S49E | 53.5 | 2.89 | 10.55 | 5.24 |
| H141F | 53.0 | 2.94 | 12.07 | 6.00 |
| E157F | 52.4 | 3.07 | 29.07 | 14.45 |
| G15W | 52.3 | 3.07 | 10.55 | 5.24 |
| L156Y | 51.3 | 2.62 | 6.02 | 3.00 |
| E139V | 51.3 | 2.73 | 24.80 | 12.31 |
| D87P | 51.0 | 2.88 | 25.73 | 13.18 |
| G43P | 51.0 | 2.83 | 10.07 | 5.02 |
| W74F | 50.5 | 2.96 | 3.44 | 1.71 |
| G67H | 48.1 | 2.65 | 17.20 | 8.57 |
| A6I | 47.2 | 3.05 | 19.66 | 9.79 |

Units: *T*_m_: °C , C_m_: M, *k*_cat_: s^−1^, *k*_cat_∕*K*_M_ : s^−1^ μM^−1^
