# Supplementary material for: Thermal Stabilization of Dihydrofolate Reductase Using Monte Carlo Unfolding Simulations and Its Functional Consequences
Source: PLoS Comput Biol. 2015 Apr 23;11(4):e1004207. doi: 10.1371/journal.pcbi.1004207 (PMC4407897; doi:10.1371/journal.pcbi.1004207)
Supplement: S3 Table — (DOCX) [file pcbi.1004207.s012.docx]

| Mutations | Delta Real Tm | MCPU | Eris | Foldx | Popmusic | SDM | Native state energy^1^ |
| --- | --- | --- | --- | --- | --- | --- | --- |
| I155T | -8.3 | 1.325±0.003 | -0.938 | -2.631 | 1.77 | -4.34 | -5048.36 |
| V75H | -11.1 | 1.346±0.004 | 8.274 | 15.727 | 1.25 | -1.23 | -5042.86 |
| I155A | -13.2 | 1.330±0.004 | -1.403 | 4.320 | 2.84 | -3.25 | -5023.36 |
| I115A | -5.6 | 1.338±0.004 | 5.673 | 4.688 | 3.00 | -3.25 | -5009.88 |
| I91L | -10.3 | 1.351±0.004 | -1.901 | 3.717 | 0.94 | 4.06 | -5027.14 |
| I155L | -5.9 | 1.354±0.004 | -1.843 | -0.803 | 1.10 | -0.59 | -5042.20 |
| W133F | -5.4 | 1.355±0.003 | -2.988 | -4.211 | 1.29 | -2.08 | -5035.31 |
| V88I | -7.4 | 1.353±0.004 | -1.513 | 0.542 | 0.46 | 0.01 | -5023.76 |
| I61V | 1.7 | 1.353±0.004 | -6.574 | 2.200 | 1.09 | -0.83 | -5023.60 |
| V40A | -8.5 | 1.357±0.003 | 3.523 | 2.652 | 3.01 | -2.73 | -5009.27 |
| I115V | -0.3 | 1.355±0.004 | -3.725 | 4.488 | 1.33 | -0.83 | -5023.28 |
| V75I | -12.5 | 1.354±0.003 | -1.026 | -3.107 | 0.71 | 0.03 | -4994.19 |
| A145T | -0.4 | 1.355±0.004 | -4.001 | 2.247 | -0.03 | 0.01 | -5041.64 |
| Wild Type | 0.0 | 1.358±0.004 | 0.000 | 0.000 | 0.00 | 0.00 | -5015.81 |
| I91V | -2.6 | 1.365±0.004 | 0.820 | 3.261 | 1.00 | 4.01 | -5014.36 |
| L112V | -4.3 | 1.374±0.004 | -0.567 | 4.167 | 1.16 | -0.39 | -4999.38 |
| D27F | 7.6 | 1.379±0.005 | -3.814 | 1.505 | -0.67 | 0.78 | -4940.97 |
| T113V | 3.9 | 1.389±0.004 | -3.439 | 2.732 | -0.41 | 1.96 | -4975.99 |
| Q108D | 1.6 | 1.361±0.004 | -3.495 | 7.162 | 0.05 | -0.76 | -5053.76 |
| S138Y | 1.5 | 1.366±0.004 | 4.594 | 2.399 | 0.45 | 2.01 | -5039.41 |
| D116F | 1.4 | 1.369±0.004 | -0.906 | -6.134 | 0.13 | 0.75 | -4986.76 |
| T68N | 1.4 | 1.367±0.003 | 0.188 | -2.595 | 0.15 | 0.39 | -5051.05 |
| E120P | 1.2 | 1.371±0.004 | -1.138 | -5.630 | 0.34 | 0.98 | -4944.57 |
| V119F | 0.8 | 1.372±0.004 | 3.447 | -8.990 | 0.86 | -0.65 | -5006.80 |
| S135I | 0.7 | 1.371±0.004 | -3.534 | -2.987 | 0.38 | 3.02 | -5023.21 |
| C152I | 0.1 | 1.377±0.004 | -11.375 | 4.404 | 0.76 | 0.32 | -5015.48 |
| H114R | 0.0 | 1.366±0.004 | 10.490 | -3.223 | 0.57 | -0.30 | -5297.58 |
| S49E | -0.6 | 1.370±0.004 | -2.037 | 6.027 | 0.56 | 2.41 | -5079.01 |
| H141F | -1.1 | 1.384±0.004 | -1.518 | 2.464 | 0.30 | 1.19 | -4978.24 |
| E157F | -1.7 | 1.385±0.003 | -1.938 | -2.778 | -0.15 | 0.77 | -4957.55 |
| G15W | -1.8 | 1.372±0.004 | 22.528 | -1.411 | 0.29 | 3.02 | -5036.44 |
| L156Y | -2.8 | 1.364±0.003 | 0.432 | -4.048 | 0.29 | -1.33 | -5024.98 |
| E139V | -2.8 | 1.391±0.004 | 2.443 | 13.576 | -0.41 | 2.15 | -4906.10 |
| D87P | -3.1 | 1.367±0.004 | -1.736 | -8.577 | 0.74 | 1.59 | -4857.90 |
| G43P | -3.1 | 1.370±0.004 | 18.260 | 0.947 | 1.71 | 1.63 | -4981.99 |
| W74F | -3.6 | 1.366±0.004 | 2.663 | 0.854 | 0.90 | -1.54 | -5010.05 |
| G67H | -6.0 | 1.369±0.005 | 2.812 | 0.974 | 0.27 | 2.99 | -5023.66 |
| A6I | -6.9 | 1.383±0.003 | -0.436 | 4.508 | -0.80 | 1.00 | -5010.53 |
| T68N_Q108D_T113V_E120P_S138Y | 7.2 | 1.400±0.004 | 5.250 | -2.405 | N.D | N.D | -5037.92 |
| T113V_E120P_S138Y | 4.4 | 1.384±0.004 | 3.316 | -6.606 | N.D | N.D | -4943.96 |
| T68N_Q108D_E120P_S138Y | 2.3 | 1.377±0.003 | 1.814 | 0.653 | N.D | N.D | -5043.41 |
| T68N_Q108D | 1.7 | 1.366±0.003 | 5.291 | 1.608 | N.D | N.D | -5076.25 |
| E120P_S138Y | 1.5 | 1.371±0.004 | 2.730 | 4.375 | N.D | N.D | -4969.24 |
| *r* |  | 0.65 | -0.06 | -0.25 | -0.55 | 0.31 | 0.04 |
| *p* value |  | < 0.0001 | 0.722 | 0.112 | 0.0003 | 0.057 | 0.779 |

^1^. The Native state energy is the minimized structure energy, calculated by NAMD
